# Correction: Biophysical Analysis of *Anopheles gambiae* Leucine-Rich Repeat Proteins APL1A^1^, APL1B and APL1C and Their Interaction with LRIM1

**DOI:** 10.1371/journal.pone.0126901

**Published:** 2015-04-20

**Authors:** The PLOS ONE Staff

## Notice of Republication

This article was republished on April 10, 2015, to correct errors in Fig. 1 and Fig. 4 that were introduced during the typesetting process. The title has also been corrected to state “APL1B” rather than “APLB.” Please download this article again to view the correct version. The originally published, uncorrected article and the republished, corrected article are provided here for reference.

## Supporting Information

S1 File Originally published, uncorrected article. (PDF)

S2 File Republished corrected article. (PDF)

## References

[1] Williams M, Summers BJ, Baxter RHG (2015) Biophysical Analysis of *Anopheles gambiae* Leucine-Rich Repeat Proteins APL1A^1^, APL1B and APL1C and Their Interaction with LRIM1. PLoS ONE 10(3): e0118911 doi: 10.1371/journal.pone.0118911 25775123 10.1371/journal.pone.0118911 PMC4361550

