# Supplementary figures and images for: Biophysical Analysis of Anopheles gambiae Leucine-Rich Repeat Proteins APL1A1, APL1B and APL1C and Their Interaction with LRIM1
Source: PLoS One. 2015 Mar 16;10(3):e0118911. doi: 10.1371/journal.pone.0118911 (PMC4361550; doi:10.1371/journal.pone.0118911)

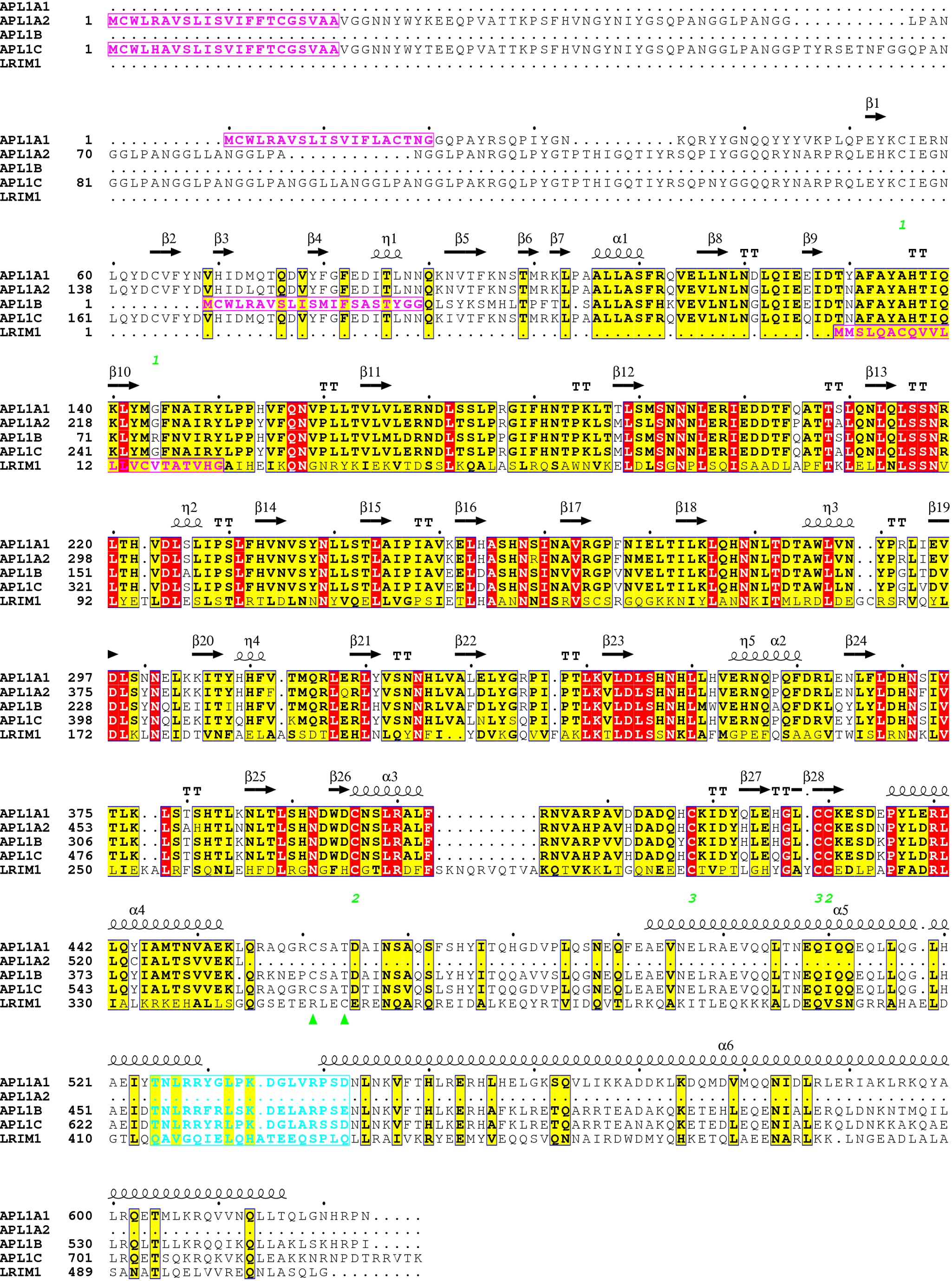

Supplement: S1 Fig — The signal peptide sequences for each are shown in purple and do not form part of the alignment. Sequences that show similarities across a group are boxed in blue, similar residues across a group are boxed and highlighted yellow, similar residues within a group are in bold and residues that are strictly conserved are highlighted in red. Symbols above blocks of sequences correspond to the secondary structure of APL1C (Chain B) of PDB ID 3OJA containing helices (grey), beta sheets (black arrows), 310-helices (η) and turns (T). HLH residues are in cyan. Green numbers below the alignment indicate the presence of disulphide bonds eg. the two number 1’s form a bond. The green arrows indicate the Cys residues involved in intermolecular disulphide bond formation between the heterodimer LRIM1/APL1C. (TIF) [file pone.0118911.s001.tif]

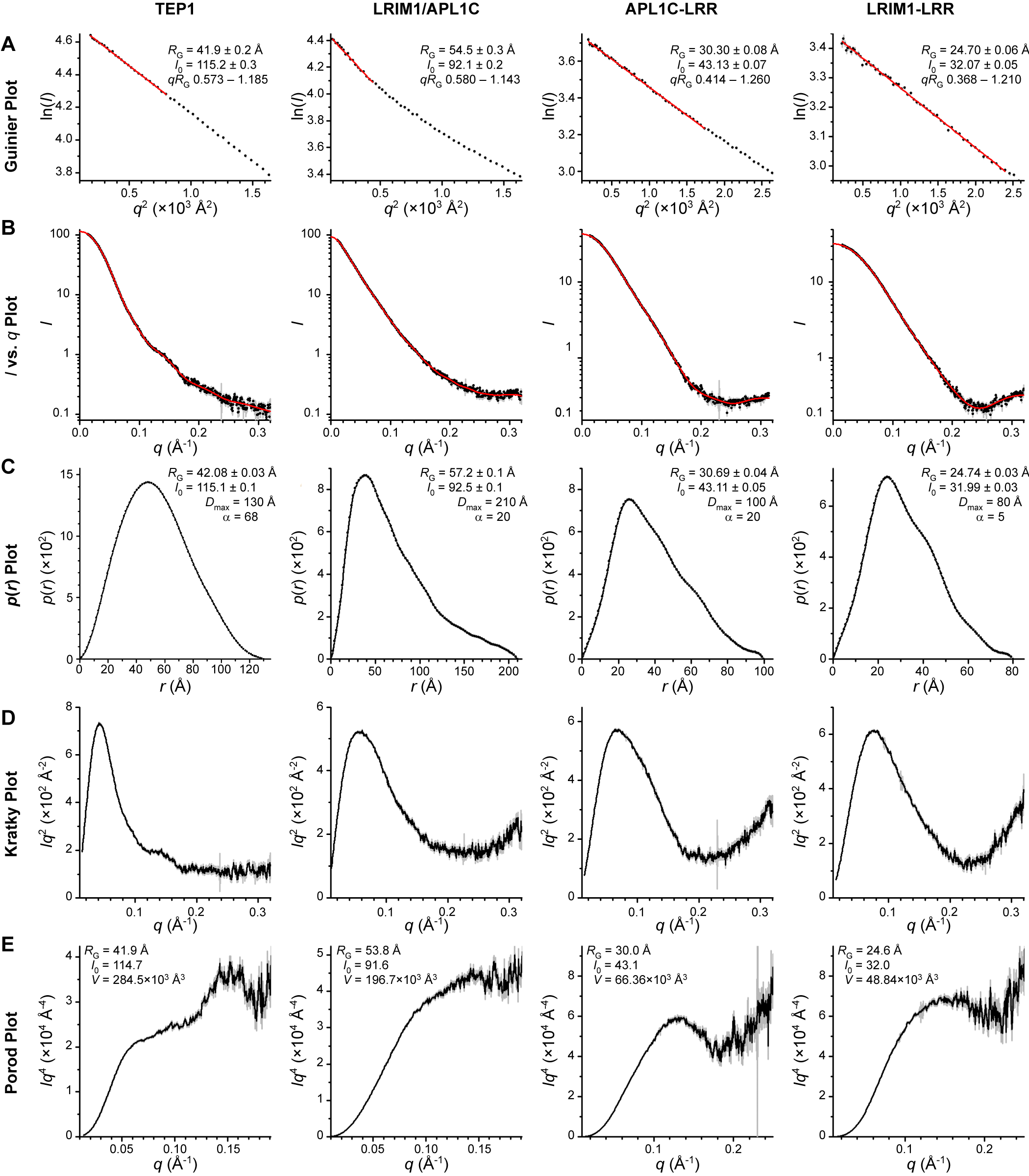

Supplement: S2 Fig — Data shown as black line or points, error as grey vertical bars, fit to data shown as red line. (A) Guinier analysis (lnI vs. q 2) with line of best fit illustrated in fitting range. (B) I vs. q curve with fit by implicit Fourier Transform (GNOM). (C) Pairwise distribution function p(r) derived by GNOM. (D) Kratky plot (Iq 2 vs. q). (E) Porod plot (Iq 4 vs. q). (TIF) [file pone.0118911.s002.tif]

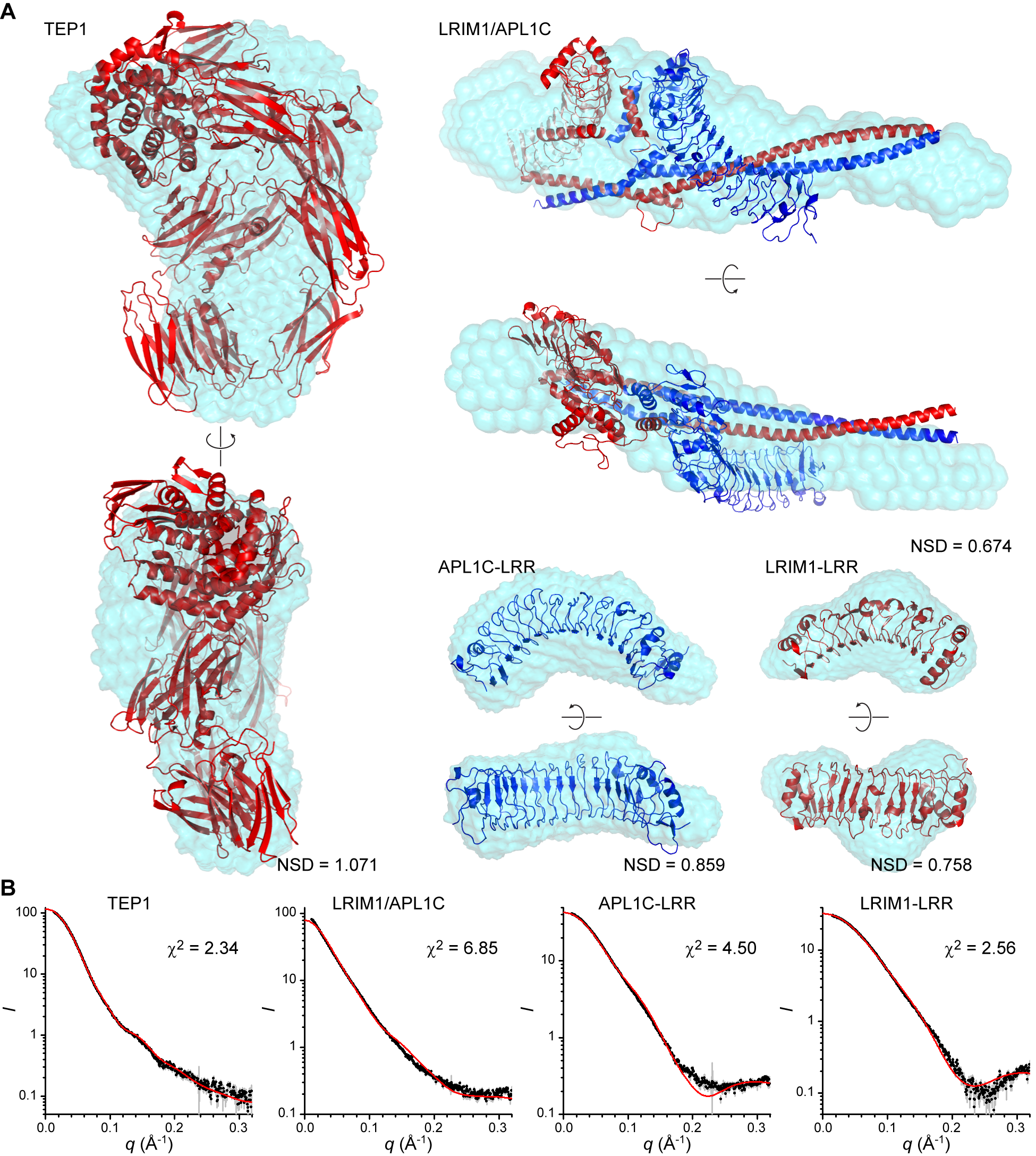

Supplement: S3 Fig — Superposition of ab initio SAXS models and best single model generated by CRYSOL for TEP1*R1, LRIM1/APL1C, APL1C-LRR and LRIM1-LRR SAXS data. (A) Bead model displayed as green surface, protein model by red/blue cartoon with CPK sticks for N-linked glycosylation. (B) Fit to experimental scattering curve for each of the static structural models shown above. (TIF) [file pone.0118911.s003.tif]

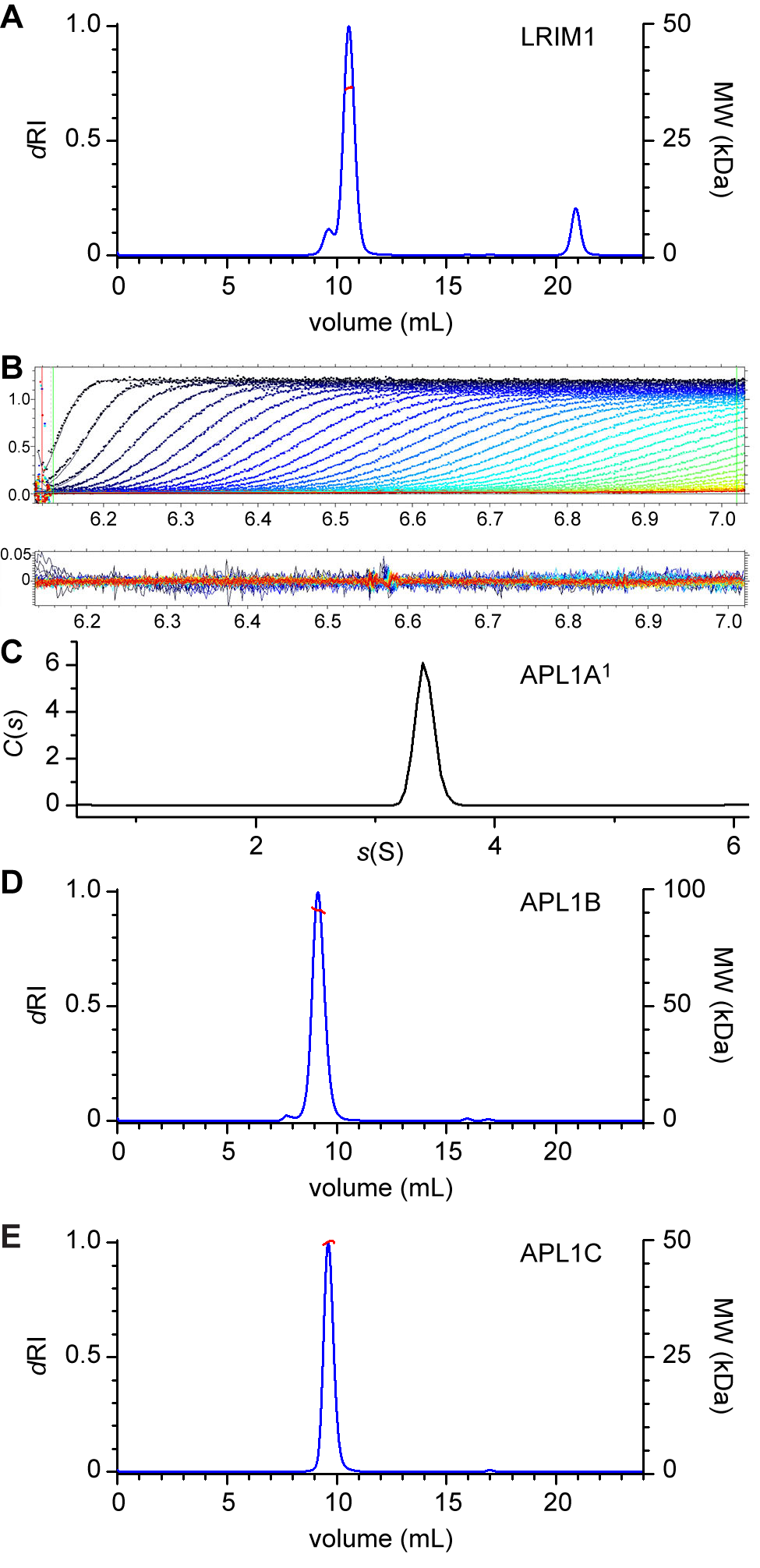

Supplement: S4 Fig — SEC-MALLS graphs show differential refactive index (dRI) on the left axis with blue trace, MW on right y axis. AUC shows fitting or raw sedimentation velocity data and transformed C(s) distribution from 0.1 < s < 6.1. (A) LRIM1-LRR (SEC-MALLS), (B-C) APL1A1 (AUC), (D) APL1B-LRR, (E) APL1C-LRR. (TIF) [file pone.0118911.s004.tif]

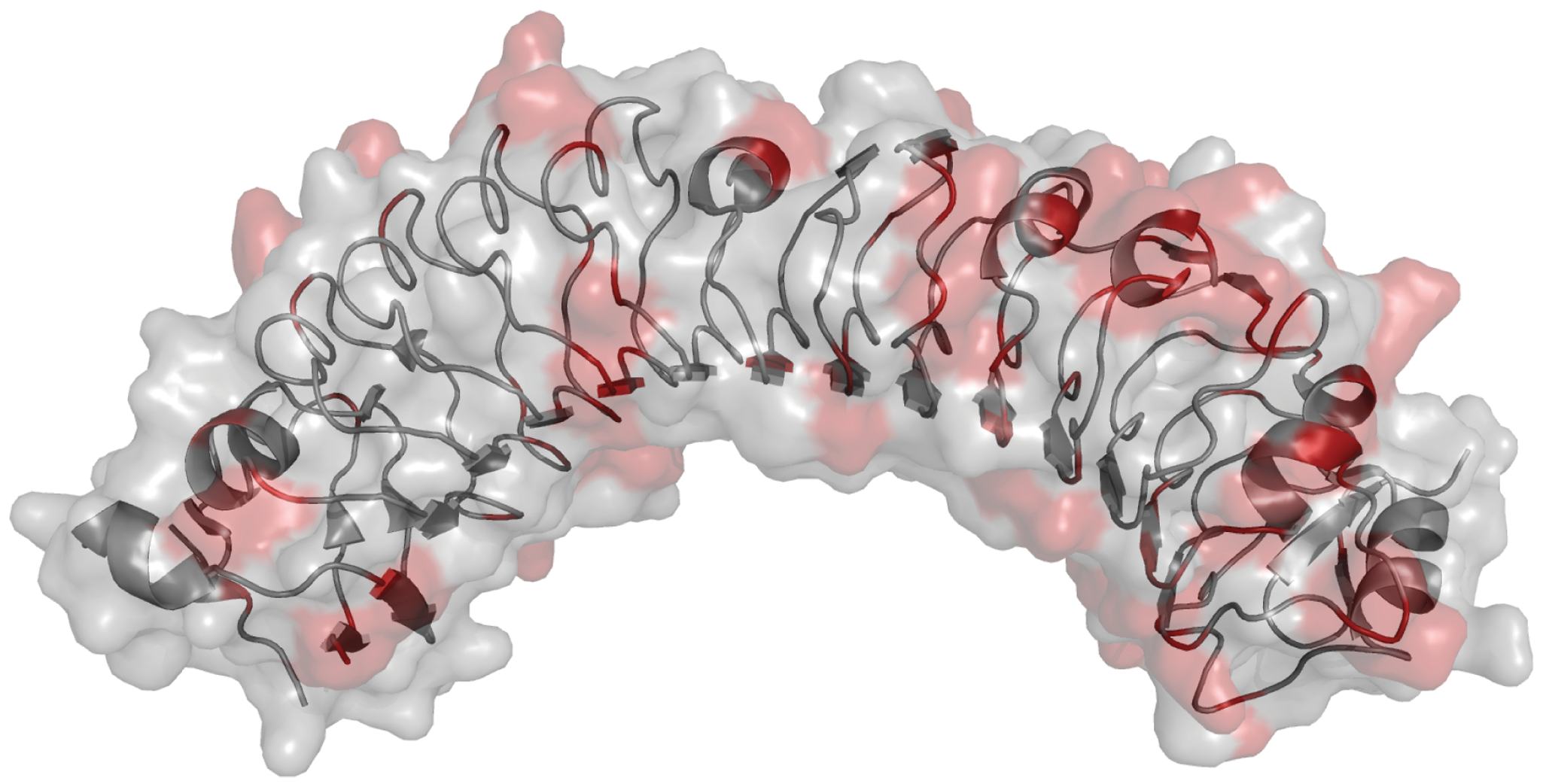

Supplement: S5 Fig — Mapping of the polymorphisms (red) between APL1A-C onto APL1C-LRR shown as a grey surface structure (PDB ID 3O6N). (TIF) [file pone.0118911.s005.tif]
