# Supplementary material for: Correction: Biophysical Analysis of Anopheles gambiae Leucine-Rich Repeat Proteins APL1A1, APL1B and APL1C and Their Interaction with LRIM1
Source: PLoS One. 2015 Apr 20;10(4):e0126901. doi: 10.1371/journal.pone.0126901 (PMC4404096; doi:10.1371/journal.pone.0126901)
Supplement: S2 File — (PDF) [file pone.0126901.s002.PDF]

RESEARCH ARTICLE

Biophysical Analysis of *Anopheles gambiae* Leucine-Rich Repeat Proteins APL1A[1], APL1B and APL1C and Their Interaction with LRIM1

Marni Williams, Brady J. Summers, Richard H. G. Baxter*

Department of Chemistry and Molecular Biophysics & Biochemistry, Yale University, New Haven, Connecticut, United States of America

* richard.baxter@yale.edu

Abstract

Natural infection of *Anopheles gambiae* by malaria-causing *Plasmodium* parasites is significantly influenced by the *APL1* genetic locus. The locus contains three closely related leucine-rich repeat (LRR) genes, *APL1A*, *APL1B* and *APL1C*. Multiple studies have reported the participation of *APL1A—C* in the immune response of *A. gambiae* to invasion by both rodent and human *Plasmodium* isolates. APL1C forms a heterodimer with the related LRR protein LRIM1 via a C-terminal coiled-coil domain that is also present in APL1A and APL1B. The LRIM1/APL1C heterodimer protects *A. gambiae* from infection by binding the complement-like protein TEP1 to form a stable and active immune complex. Here we report solution x-ray scatting data for the LRIM1/APL1C heterodimer, the oligomeric state of LRIM1/APL1 LRR domains in solution and the crystal structure of the APL1B LRR domain. The LRIM1/APL1C heterodimeric complex has a flexible and extended structure in solution. In contrast to the APL1A, APL1C and LRIM1 LRR domains, the APL1B LRR domain is a homodimer. The crystal structure of APL1B-LRR shows that the homodimer is formed by an N-terminal helix that complements for the absence of an N-terminal capping motif in APL1B, which is a unique distinction within the LRIM1/APL1 protein family. Full-length APL1A[1] and APL1B form a stable complex with LRIM1. These results support a model in which APL1A[1], APL1B and APL1C can all form an extended, flexible heterodimer with LRIM1, providing a repertoire of functional innate immune complexes to protect *A. gambiae* from a diverse array of pathogens.

OPEN ACCESS

Citation: Williams M, Summers BJ, Baxter RHG (2015) Biophysical Analysis of *Anopheles gambiae* Leucine-Rich Repeat Proteins APL1A[1], APL1B and APL1C and Their Interaction with LRIM1. PLoS ONE 10(3): e0118911. doi:10.1371/journal.pone.0118911

Academic Editor: Bostjan Kobe, University of Queensland, AUSTRALIA

Received: March 21, 2014

Accepted: January 13, 2015

Published: March 16, 2015

Copyright: © 2015 Williams et al. This is an open access article distributed under the terms of the Creative Commons Attribution License, which permits unrestricted use, distribution, and reproduction in any medium, provided the original author and source are credited.

Data Availability Statement: All structural data are available from the Protein Databank (accession number 4XGO). All other relevant data are within the paper and its Supporting Information files.

Funding: The authors have no support or funding to report.

Competing Interests: The authors have declared that no competing interests exist.

Introduction

Malaria results from infection with *Plasmodium* parasites and is exclusively transmitted by *Anopheles* mosquitoes. Despite being both curable and preventable, malaria caused an estimated 584,000 deaths in 2013, mostly African children living in poverty [1]. Prevention, especially vector control measures such as insecticide-treated bed nets (ITNs) and indoor residual insecticide spraying (IRS), dramatically reduces the malaria burden. However, the effectiveness of

vector control is threatened as malaria mosquitoes develop resistance to the insecticides used in ITNs and IRS [1]. The African mosquito vector A. gambiae possesses an immune response that is effective against various pathogens, including malaria parasites. Destruction of parasites by the mosquito's own immune system prevents their further transmission to humans [2,3]. Hence, understanding *Anopheles-Plasmodium* host-pathogen interactions and the mechanism of parasite killing within the *Anopheles* mosquito informs both the dynamics of transmission and potentially the development of new malaria control measures.

Genetic analysis of variation in natural infection of *A. gambiae* populations identified a region on chromosome 2L that is strongly linked to *P. falciparum* resistance [4–6]. Three gene paralogs named *APL1* (*Anopheles Plasmodium*-responsive Leucine-rich repeat protein 1) within an 18 kb locus were identified as resistance candidates: *APL1A*, *APL1B* and *APL1C* [7]. At least three allelic variants of *APL1A* (*APLA*^{1}, *APL1A*^{2} *and APL1A*^{3}) have been identified in A. gambiae laboratory strains from Gambia and Cameroon [7,8], and in field-caught mosquitoes from Mali [9]. The *APL1* genes are homologous in sequence, polymorphic and under positive selection pressure. The APL1 proteins contain a signal peptide, a leucine-rich repeat (LRR) domain and a cysteine-rich region followed by a C-terminal coiled-coil (CC) domain containing a helix-loop-helix (HLH) motif (Fig. 1). APL1C forms a disulfide-linked heterodimeric complex with another anti-*Plasmodium* factor LRIM1 (Leucine-Rich Immune Molecule 1) [10–12]. LRIM1 is a paralog of APL1A—C and is structurally homologous to APL1C [13]. LRIM1 and APL1A—C are members of an LRR family, the LRIM family, that includes several dozen genes found within, but not outside, mosquito genomes (family Culicidae) and are believed to play multiple roles within the innate immune system [14].

The LRIM1/APL1C complex binds and stabilizes a specific form of *A. gambiae* Thioester-containing Protein 1 (TEP1) [11,12], a structural and functional homolog of vertebrate complement C3 [15,16]. Binding of TEP1 leads to the killing of bacteria by phagocytosis [17], and lysis and melanization of *P. berghei* parasites [2,18,19]. A strong association has been shown between *TEP1* and resistance to *P. falciparum* in refractory strains of *A. gambiae* [20,21]. Proteolytic processing of TEP1 within a protease sensitive region is required to produce a cleaved form ($TEP1_{cut}$) that is responsible for the initial attachment to pathogen surfaces. However, $TEP1_{cut}$ is an unstable species that precipitates over time in the absence of the LRIM1/APL1C complex [11,13].

The anti-*Plasmodium* phenotype of APL1A—C varies depending on *A. gambiae* strain and *Plasmodium* species or isolate used. Studies in the G3 (susceptible) and L3–5 (refractory)

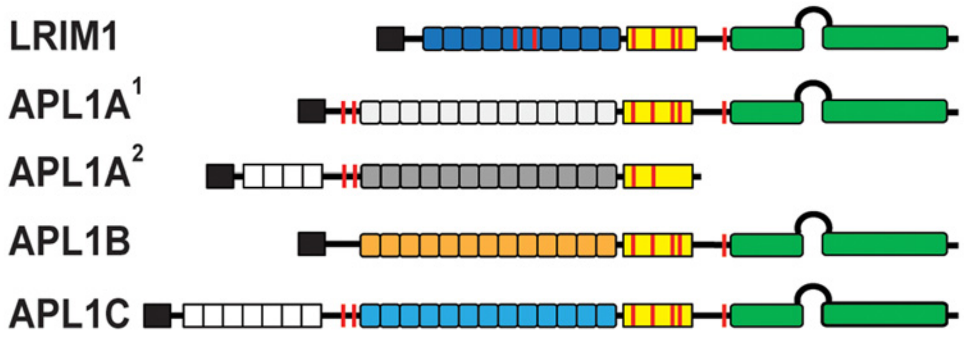

Fig 1. Schematic diagram of the LRIM1 and APL1 proteins. Colored boxes: black, signal peptide; white, low-complexity PANGGL region; yellow, Cys-rich region; green, coiled-coil (CC) domain. Boxes represent the number of LRR repeats for each protein. Features: loop between CC domains, helix-loop-helix (HLH) region; red line, Cys residue.

doi:10.1371/journal.pone.0118911.g001

strains of *A. gambiae* [22] knocking down either LRIM1 (*dsLRIM1*)or all APL1 paralogs (*dswAPL1*) demonstrated a role in the immune response to the rodent malaria parasite *P. berghei* [5,10–12]. Subsequently, only *dsAPL1C* demonstrated a phenotype for *P. berghei* infection in G3 [7]. Using the recently colonized the Ngousso from Cameroon [23], a *dswAPL1* phenotype was observed against *P. falciparum* isolates resulting from natural infection, but only *dsAPL1A* had a phenotype against the cultured *P. falciparum* isolate NF54 while only *dsAPL1C* demonstrated a phenotype against *P. berghei* or *P. yoelii* [24]. A further analysis of the Ngousso/NF54 infection model suggested the phenotype was specifically due to the *APL1A[2]* allele, which lacks the C-terminal CC domain and is not constitutively secreted from cells [8]. Studies using an outbred strain, Keele [25], and *P. falciparum* NF54 had different outcomes depending on infection intensity: a phenotype was only observed at low or medium infection intensities for *dswAPL1*, and specifically for *dsAPL1B* or *dsAPL1C*, but not *dsAPL1A* [26]. The NF54 isolate is able to infect the otherwise refractory A. gambiae L3–5 strain by evading the TEP1 immune response [21,27]; such adaptation may partly explain the variable phenotype of APL1 knockdown.

The majority of the sequence variation between the APL1 proteins exists at the N- and C-termini of the protein sequences (S1 Fig.). APL1A[2] and APL1C contain an N-terminal low-complexity region of variable extent (22–77 amino acids in APL1C) with multiple (P,L)–-ANGG–(P,L) repeats [9]. APL1A and APL1C each contain 15 LRR repeats, APL1B has 13 and LRIM1 only has 11 [14]. Allele specific differences in *APL1A[2]* and *APL1A[3]* result in premature stop codons upstream of the CC domains. Within the CC domain, APL1B is significantly different from both APL1A and APL1C in the HLH motif. Outside of the HLH, APL1B and APL1C are identical within the CC region except for the last 30 residues, while APL1A[1] diverges from APL1C in the last 60 residues (S1 Fig.).

Extracellular LRR proteins are typically flanked by disulfide-containing capping motifs at the N-and C-termini, called LRRNT and LRRCT, respectively, that protect the hydrophobic cores of the first and last LRRs [28]. The LRIM family has a unique LRRCT that distinguishes it from other LRR families [14]. The LRRCT motif has two disulfide bonds with a conserved structure that is observed in the crystal structures of LRIM1 and APL1C [13]. LRIM1 and APL1C also form an intermolecular disulfide bridge between LRIM1 Cys352 and APL1C Cys562. The latter cysteine is conserved in APL1B and APL1A[1] but not in APL1A[2] (S1 Fig.). The APL1 LRRNT capping motif contains only a single disulfide bond that was resolved in the crystal structure of the APL1C LRR domain (PDB ID 3O6N). While this motif is found in almost all members of the LRIM family, APL1B and LRIM1 do not have a defined LRRNT, which is unusual for extracellular LRR proteins (S1 Fig.).

We have previously hypothesized that the LRR domains of LRIM1/APL1 act as molecular spacers [13], creating a defined distance between the N-terminal and C-terminal variable regions that are the primary binding sites for other immune factors. For instance, the CC domain and the HLH motif in particular, appear crucial for binding and stabilization of $TEP1_{cut}$ [13,29]. However, while APL1A[1] and APL1B possess CC domains similar to APL1C it is not known whether these proteins form similar complexes with LRIM1, and if so, whether that complex interacts with TEP1 or other *A. gambiae* TEPs. The answer to this question directly impacts our understanding of the unknown mechanism of APL1A's observed phenotype against human malaria, and whether multiple TEP1-mediated or TEP1-independent mechanisms of *Plasmodium* killing operate in *A. gambiae*. Furthermore, the absence of the LRRNT motif in APL1B raises the question as to how the N-terminus of the LRR fold is stabilized and whether this has any relevance to its unknown function.

Here we report further *in vitro* studies of the APL1A and APL1B proteins. The structure of the LRIM1/APL1C heterodimer is extended and flexible in solution. Structural analysis of

APL1B identified that the protein exists as a homodimer that is mediated by a unique N-terminus which has no homology to other LRIM family members [14]. Finally, we show that APL1B forms a disulfide-bridged heterodimer with LRIM1 analogous to APL1C. These results are likely to have direct bearing on the functional role of APL1B in the *A. gambiae* immune system.

Results

The crystal structure of LRIM1/APL1C does not represent its structure in solution

The crystal structures of TEP1 (both *R1 and *S1 alleles), the LRR domains of LRIM1 (LRIM1-LRR) and APL1C (APL1C-LRR), and the LRIM1/APL1C heterodimer have been previously determined. To test whether these structures were representative of the structure in solution we analyzed TEP1*R1, LRIM1/APL1C, APL1C-LRR and LRIM1-LRR using small-angle x-ray scattering (SAXS). The proteins behaved as homogeneous, monodisperse solutions within the concentration range 1–5 mg/ml (supplementary methods). Primary analysis of the Guinier plot, $p(r)$ distribution, Kratky and Porod plots further confirmed that the samples are generally folded, monodisperse particles (S2 Fig.). A consistent measure of I_0 and R_G were determined for all samples (Table 1) with one exception; the R_G for LRIM1/APL1C derived from the $p(r)$ distribution (R_G = 57.2 ± 0.1 Å) was significantly greater than that determined by Guinier analysis (R_G = 54.5 ± 0.3 Å). The LRIM1/APL1C $p(r)$ distribution is also the function with the lowest confidence score in *GNOM* [30], likely due to the functions extended tail at large interatomic distances up to D_{max} = 210 Å. While consistent with the long rod-like feature of the LRIM1/APL1C coiled-coil, this makes D_{max} difficult to determine. Indeed a function with the same I_0 can be produced up to D_{max} = 250 Å, which is the maximum D_{max} for the implicit Fourier transform given the sampling limit set by smallest q value measured in the experiment.

Table 1. Parameters derived by primary analysis of SAXS data.

TEP1	LRIM1/APL1C	APL1C-LRR	LRIM1-LRR
Guinier Analysis (*PRIMUS*)			
R_G = 41.9 ± 0.2 Å	R_G = 54.5 ± 0.3 Å	R_G = 30.30 ± 0.08 Å	R_G = 24.70 ± 0.06 Å
I_0 = 115.2 ± 0.3	I_0 = 92.1 ± 0.2	I_0 = 43.13 ± 0.07	I_0 = 32.07 ± 0.05
qR_G = 0.573–1.185	qR_G = 0.580–1.143	qR_G = 0.414–1.260	qR_G = 0.368–1.210
$p(r)$ distribution (*GNOM*)			
R_G = 42.08 ± 0.03 Å	R_G = 57.2 ± 0.1 Å	R_G = 30.69 ± 0.04 Å	R_G = 24.74 ± 0.03 Å
I_0 = 115.1 ± 0.1	I_0 = 92.5 ± 0.2	I_0 = 43.11 ± 0.05	I_0 = 31.99 ± 0.03
D_{max} = 130 Å, α = 68	D_{max} = 210 Å, α = 20	D_{max} = 100 Å, α = 20	D_{max} = 80 Å, α = 5
Total score 0.738	Total score 0.482	Total score 0.681	Total score 0.690
Porod Analysis (*PRIMUS*)			
R_G = 41.9 Å	R_G = 53.8 Å	R_G = 30.0 Å	R_G = 24.6 Å
I_0 = 114.7	I_0 = 91.6	I_0 = 43.1	I_0 = 32.0
V = 284,500 Å^3	V = 196,700 Å^3	V = 66,360 Å^3	V = 48,840 Å^3
B_{est} = 0.074	B_{est} = 0.08	B_{est} = 0.06	B_{est} = 0.005
Crystal structure (*CRYSOL*)			
R_G = 42.5 Å	R_G = 45.0 Å	R_G = 30.2 Å	R_G = 25.1 Å
χ^2 = 2.34	χ^2 = 6.85	χ^2 = 4.50	χ^2 = 2.55

doi:10.1371/journal.pone.0118911.t001

A set of twenty *ab initio* bead models were generated by *DAMMIF* [31] for each structure and were generally self-consistent as judged by their normalized structural discrepancy (NSD). The visual fit of the bead models (S3A Fig.) for TEP1*R1 and APL1C-LRR were quite reasonable except for the hole in the β-ring of TEP1 which is counter to one of the assumptions in *ab initio* models, hence the volume is narrower in that region. For LRIM1-LRR the fit was reasonable but with extra unaccounted volume in the bead model. For LRIM1/APL1C however, the fit was poor. The bead model is essentially a tube that is significantly longer than the crystal structure and, despite thickening at one end, the model does not accommodate the two LRR domains; the ends project out of the volume as does the end of the CC region.

Two approaches were used to compare the known crystal structures of each domain to the solution scattering curve. First, we used the program *CRYSOL* [32] to calculate the fit of the crystal structure plus a hydration shell to the scattering curve (S3B Fig.). The goodness-of-fit as judged by the χ^2 statistic were reasonable for TEP1*R1 and LRIM1-LRR; less so for APL1C due to deviations at $q \sim 0.2$ Å^{-1} values, but the R_G of each hydrated crystal structure was similar to the experimental value (Table 1). For LRIM1/APL1C however, the fit was appreciably worse ($\chi^2 = 6.85$), and notably the R_G of the hydrated crystal structure ($R_G = 45.0$ Å) was significantly less than the experimental value.

Two sources of error in comparing the crystal structures to solution scattering curves is (i) missing N- and C-terminal residues and internal loops that are disordered in the crystal structure, and (ii) *N*-linked glycosylation which is disordered and also affects the predicted scattering from the hydration shell surrounding the protein. To model these additional factors we used the software *ALLOSMOD-FOXS* [33] to generate a series of static models containing multiple conformations of both protein loops and *N*-linked glycans (Fig. 2A). For TEP1*R1, APL1C-LRR and LRIM1-LRR this led to a significant improvement in the goodness-of-fit, as judged by the χ^2statistic, for a single model (Fig. 2B). For LRIM1/APL1C however, the fit remains poor ($\chi^2 = 5.14$), and the model $R_G = 47.5$ Å is still significantly below the experimental value.

Taken together, these results suggest that a single glycosylated conformation based on the crystal structure is consistent with the observed solution scattering curve for TEP1*R1, LRIM1-LRR and APL1C-LRR. Hence, the solution structure is most likely a local ensemble of similar structures as the single best conformation. For LRIM1/APL1C however, the experimental scattering curve cannot be accurately modeled by a conformation based upon the known crystal structure. The experimental evidence suggests the structure in solution is more extended, which requires movement of either or both of the LRR domains away from the CC domain. A satisfactory minimal ensemble of structures for LRIM1/APL1C that fit the experimental curve has not yet been derived by flexible fitting approaches.

The LRR domain of APL1B forms a homodimer in solution

We then examined the state of the APL1A[1] and APL1B LRR domains in solution in comparison to APL1C and LRIM1. The four LRRs were expressed in insect cells, purified to homogeneity and analyzed by size-exclusion chromatography (SEC) (Fig. 3). As expected from their been prior purification [13] and SAXS analysis, LRIM1-LRR and APL1C elute at an apparent molecular weight consistent with a monomer (Table 2). APL1A[1]-LRR includes a short extension at the N-terminus compared to APL1C-LRR. Partial proteolysis in this region was observed in purified APL1A[1]-LRR, which appears as a doublet on SDS-PAGE (Fig. 3, inset). The N-terminal cleavage site was determined by Edman sequencing to occur at position Gln 42. Nevertheless, APL1A[1]-LRR also elutes at an apparent molecular weight of a monomer. Surprisingly

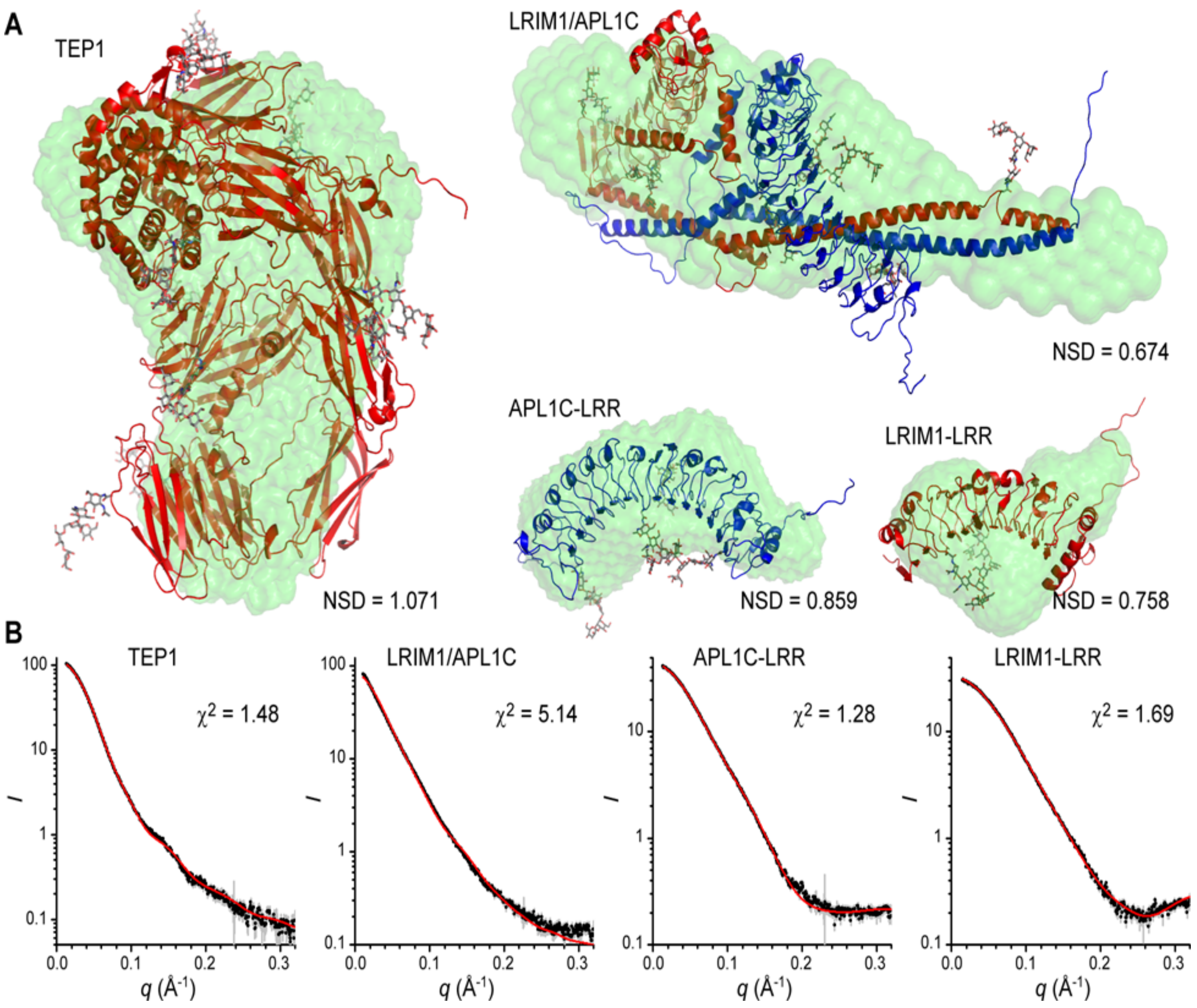

Fig 2. SAXS modeling of TEP1*R1, LRIM1-LRR, APL1C-LRR and LRIM1/APL1C. Superposition of *ab initio* SAXS models and best single model generated by *ALLOSMOD-FOXS* for TEP1*R1, LRIM1/APL1C, APL1C-LRR and LRIM1-LRR SAXS data. (A) Bead model displayed as green surface, protein model by red/blue cartoon with CPK sticks for N-linked glycosylation. (B) Fit to experimental scattering curve for each of the static structural models shown above.

doi:10.1371/journal.pone.0118911.g002

however, APL1B-LRR eluted at a higher apparent molecular weight (MW ~90 kDa), consistent with a dimer.

Retention on SEC is related to a protein's hydrodynamic radius, which is influenced by shape as well as molecular weight (MW). We therefore used SEC with multi-angle laser light scattering (SEC-MALLS) or analytical ultracentrifugation (AUC) to verify the apparent MW of each protein from SEC (Table 2, S4 Fig.). The MW determined for APL1B-LRR was 91 kDa, almost twice that measured for APL1A[1]-LRR (55 kDa), APL1C-LRR (50 kDa) and LRIM1-LRR (36 kDa). The MW of the monomers are several kDa greater than calculated due to N-linked glycosylation (APL1A[1]–3 sites, APL1B—3 sites, APL1C—4 sites, LRIM1–2 sites). Addition of EDTA to the buffer did not dissociate the APL1B-LRR dimer, ruling out metal-induced aggregation via the C-terminal 6×His tag. Since all APL1 LRRs are highly homologous in sequence, we concluded that the dimeric state of APL1B-LRR is due to its unique N-terminal sequence.

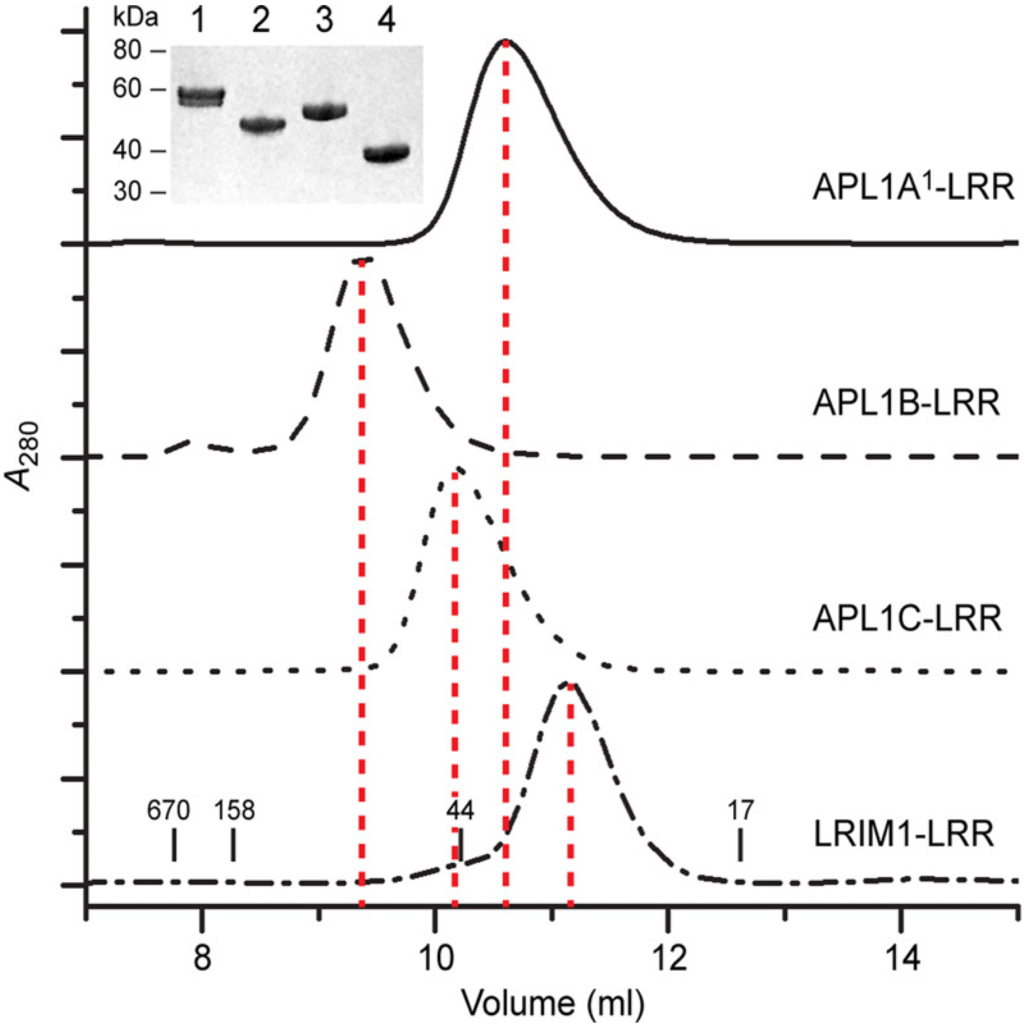

Fig 3. Solution State of LRIM1 and APL1A—C LRR domains. Solution MW determination of the LRR domains of APL1A[1], APL1B, APL1C and LRIM1 by SEC. The retention volumes of molecular mass standards in kDa are indicated on the bottom panel. (Inset) Purity of each protein on SDS-PAGE, lanes: (1) APL1A[1], (2) APL1B, (3) APL1C and (4) LRIM1.

doi:10.1371/journal.pone.0118911.g003

Table 2. Molecular weight of LRIM1/APL1 LRR domains in solution.

LRR domain	MW_{calc} (kDa)[a]	MW_{exp} (kDa)
LRIM	35.6	36.4[b]
APL1A[1]	49.5	55[c]
APL1B	41.1	91.1[b]
APL1C	47.3	49.7[b]

[a]Calculated monomeric mass, excluding glycosylation

[b]As measured by multi-angle laser light scattering (MALLS)

[c]As measured by analytical ultracentrifugation (AUC)

doi:10.1371/journal.pone.0118911.t002

Crystal structure of the APL1B-LRR homodimer

To determine the molecular basis of APL1B-LRR homodimerization, we crystallized APL1B-LRR and solved the structure to 1.74 Å resolution (Table 3). The refined model contains two molecules of APL1B-LRR in the asymmetric unit, residues 25–364 for chain A and residues 27–364 for chain B (the crystallized protein comprises APL1B 21–370 and a C-terminal 6×His tag). Of the three predicted *N*-linked glycosylation sites (Asn166, Asn211, Asn317) a single *N*-acetylglucosamine was resolved for residue Asn166 of each domain. The overall structure of APL1B-LRR is similar to APL1C-LRR (Cα rmsd 0.605 Å). However, APL1B has two fewer LRRs than APL1C so the overall length of its LRR domain is ~70 Å versus ~80 Å for APL1C (Fig. 4A). The N-terminus of APL1B is well resolved (Fig. 4B). The LRRCT motif is conserved with APL1C (Fig. 4C).

Table 3. Data collection and refinement statistics for the crystal structure of APL1B-LRR.

**Data Collection**	
Beam line	NSLS X25C
Wavelength (Å)	1.1
Space Group	$P2_12_12_1$
Unit cell (*a*; *b*; *c*) (Å)	64.423; 74.956; 214.370
Resolution (Å)	50.0–1.74
Unique reflections	102271 (4549)
Redundancy	6.1 (4.4)
Completeness (%)	96.1 (86.7)
$\langle I \rangle / \langle \sigma \rangle$	36.7 (2.6)
R_{sym} (%)	4.3 (53)
**Refinement**	
Resolution (Å)	40.36–1.74 (1.79–1.74)
Reflections	
Working set	91036 (3753)
Test set	4796 (226)
R_{cryst} (%)	18.7 (23.6)
R_{free} (%)	22.0 (25.4)
ESU (MLF) (Å)	0.071
RMSD from ideal	
Bond lengths (Å)	0.008
Bond angles (°)	1.314
Avg. B-factor (Å^2)	17.9
Atoms	6299
protein	5400
Heteroatom	118
water	781
Molprobity analysis	
All-atom clash score	3.48
Bad rotamers (%)	1.31
Ramachandran plot	
Outliers (%)	0.3 (2/676)
Favored (%)	92.6 (626/676)

doi:10.1371/journal.pone.0118911.t003

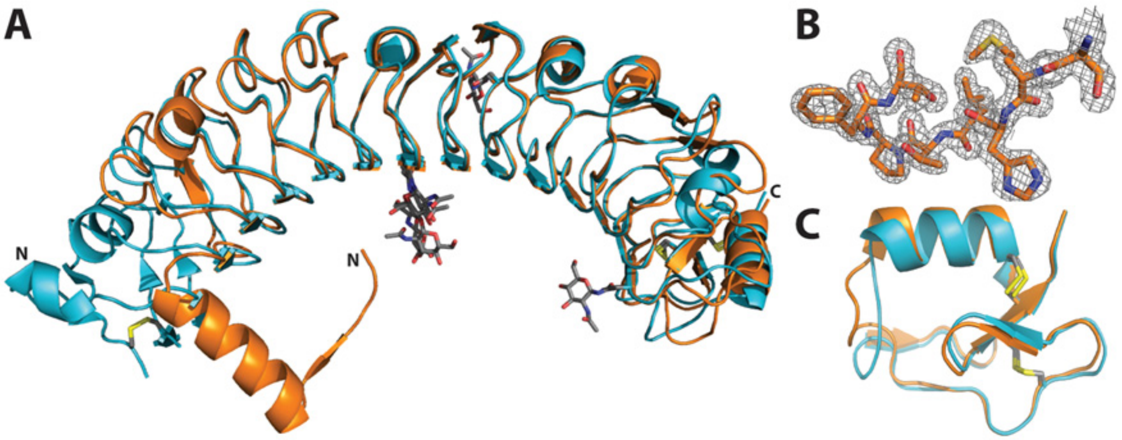

Fig 4. Crystal structure of the LRR domain of APL1B. A: Alignment of the LRR domains of APL1C (PDB ID 3ON6, turquoise) and APL1B (this study, orange). N-linked glycosylation sites and disulfide bonds are shown as sticks (CPK coloring). The N- and C-termini are indicated. B: Example of $2F_o–F_c$ density map at the N-terminus of residues 27–34 of APL1B at 1.5σ. C: Alignment of the LRRCTs of APL1C and APL1B. The disulfide bonds are shown as grey and yellow sticks.

doi:10.1371/journal.pone.0118911.g004

A homodimeric interface exists between the two APL1B-LRR domains in the asymmetric unit that buries an area of ~1500 Å^2 representing 9–10% of total solvent-accessible surface area (Fig. 5A). The dimer interface is formed between the N-terminal helix of one domain and the first LRR of the other domain. The two N-terminal helices form a dovetail joint between the solenoid LRRs that hold the two domains at a 90° angle to each other. Residues 31–44 form an amphipathic α-helix such that Leu35, Leu38, Leu39, and Phe42 pack against the first LRR, protecting the hydrophobic surface from the solvent (Fig. 5B). Interestingly, a short, well resolved β-strand on the N-terminal loop of one domain (Met28–Leu30) forms a parallel β-sheet with a β-strand on the first LRR of the second domain (Ile55–Ile58). Backbone interactions are formed between residues Met28 and Leu30 with Gln54, Glu57 and Asp59 on the second domain (Fig. 5C).

We compared the structure of the N-terminus of APL1B to that of APL1C and LRIM1. Several differences exist at the N-termini of the LRR domains. In the absence of an LRRNT, APL1B has an α-helix that protects the first LRR of the second domain within the homodimer (Fig. 5D). In contrast, APL1A and APL1C cap the leading LRR by a canonical LRRNT (Fig. 5E), while a short antiparallel β-strand and an α-helix formed by residues 43–58 protect LRIM1's leading LRR (Fig. 5F).

We compared the specific APL1B residues located at the homodimeric interface with the corresponding residues in APL1C by sequence alignment (Fig. 6). Intriguingly, we found that the secondary structure elements of the APL1B N-terminus are found on the convex face of LRR II of APL1C and LRR I of APL1B is identical to the corresponding LRR III of APL1C (shown in yellow in Figs. 4D, E and Fig. 6). Hence, an N-terminal truncation in the middle of LRR II leads APL1B to form a homodimer to protect the hydrophobic face of the leading LRR via domain swapping with secondary structure complementation [34].

The *APL1* genes are highly polymorphic and under positive selection pressure, presumably based on the binding of either other *Anopheles* proteins or of *Plasmodium* or other microbial ligands. If a specific portion surface of the LRR domain were responsible for binding a common ligand (e.g. another Anopheles immune factor) we may expect the residues within that region would be conserved between APL1 paralogs. Conversely, if a specific region on the surface of

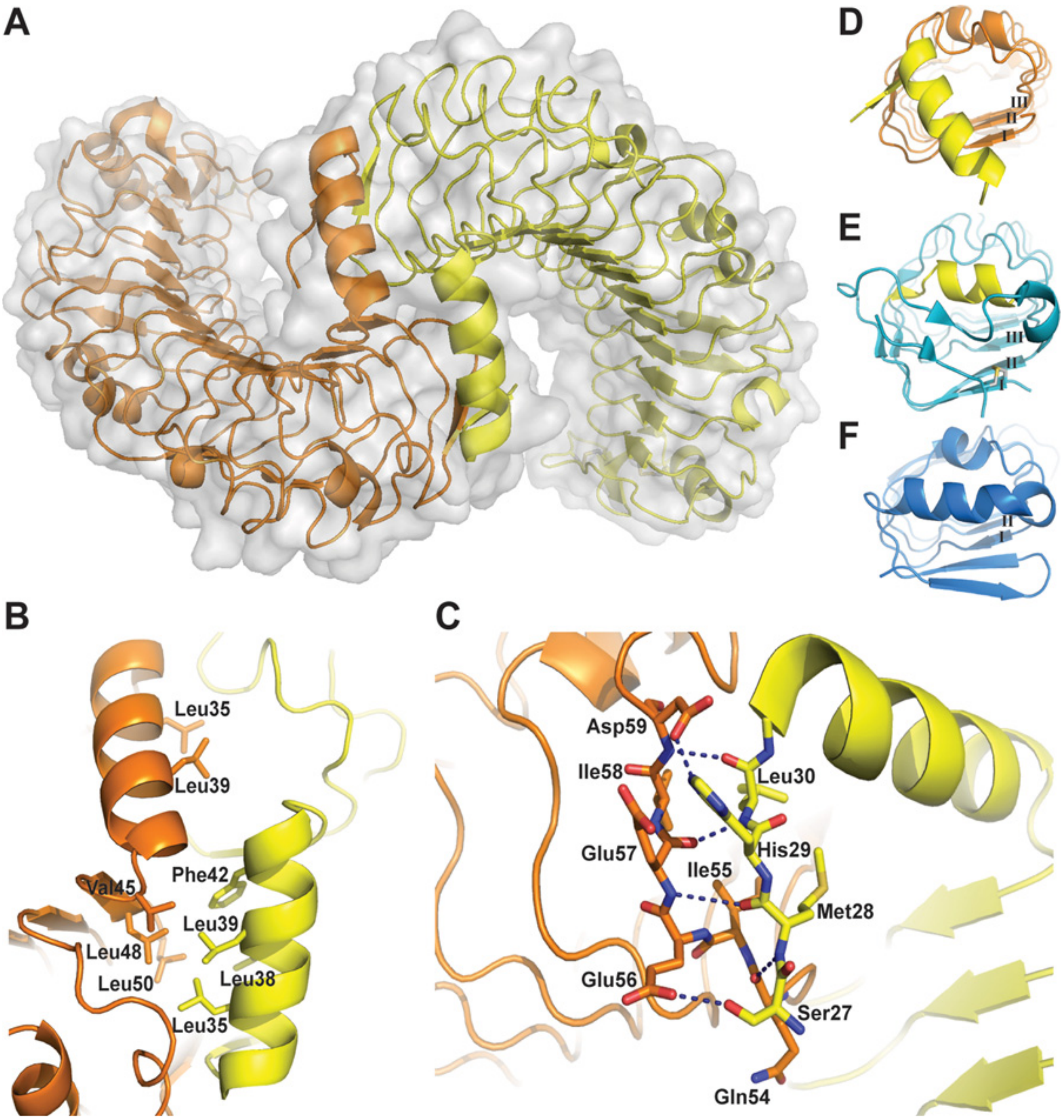

Fig 5. The dimerization interface of APL1B-LRR. A: Surface maps of the two APL1B-LRR molecules in the asymmetric unit showing N-terminal linked dimerization of the domains. B: Hydrophobic residues on the amphipathic N-terminal α-helix are protected from the solvent by a second APL1B-LRR domain. The hydrophobic residues (Val45, Leu48, Leu50) on the first LRR that are also protected by this interaction are shown. C: Backbone β-sheet interactions between two parallel β-strands (Met28–Leu30 and Ile55–Ile58) of the two APL1B-LRR domains that mediate the N-terminal dimerization interface. Electrostatic interactions between Ser27–Gln54 and His29–Asp59 are also shown. Hydrogen bonds are shown as dark blue dashes. Crystal structures of (D) APL1B-LRR, (E) APL1C-LRR and (F) LRIM1-LRR looking down the solenoid from the N-terminus. The secondary structural elements that are similar between the N-terminus of APL1B (helix from second chain) and the convex face of LRR II of APL1C are highlighted in yellow. Individual LRRs are numbered.

doi:10.1371/journal.pone.0118911.g005

the LRR domain were a site of selection for binding different ligands (e.g. from different *Plasmodium* species), then mapping polymorphisms within or between APL1A-C onto either the APL1B-LRR or APL1C-LRR structure might reveal a localized hypervariable patch on the surface, as certain hypervariable loops between TEP1 alleles are proximal to the thioester bond [15,16]. Mapping of polymorphisms between APL1A—C onto the APL1C-LRR crystal

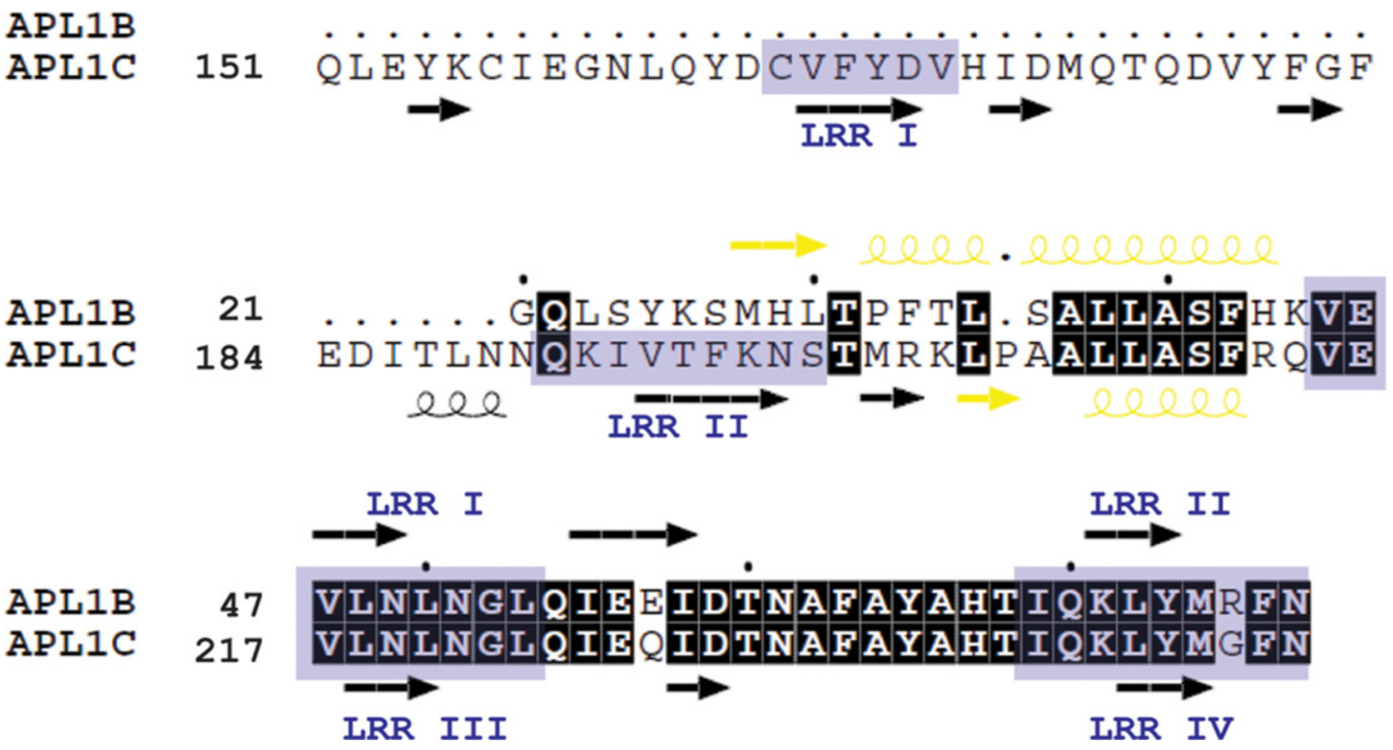

Fig 6. Alignment of the N-termini of APL1B and APL1C. Conserved residues are highlighted in black. The secondary structural elements that are similar between the N-terminus of APL1B and the convex face of LRR II of APL1C are highlighted in yellow. The LRRs are numbered and shown in grey boxes for APL1B and APL1C. The secondary structure elements of APL1B (PDB ID 4XGO) and APL1C (PDB ID 3O6N) are shown as coils for α-helices and arrows for β-strands.

doi:10.1371/journal.pone.0118911.g006

structure (PDB ID 3O6N) however, shows that the mutations are distributed all over the concave and convex faces of the structure (S5 Fig.). This is consistent with the hypothesis that the LRR domain itself is not the primary site of interaction between LRIM1, APL1A—C and their (unknown) ligands.

Heterodimerization of APL1A—C with LRIM1

Given the similarities between APL1A[1], APL1B and APL1C, we hypothesized that APL1A[1] and APL1B may form a heterodimer with LRIM1 as seen for APL1C [13]. We tested this hypothesis by generating recombinant baculovirus for dual-expression of 6×His-tagged LRIM1, APL1A[1], APL1A[2] APL1B, and APL1C with FLAG-tagged LRIM1 (Fig. 7A) in *T. ni* cells and performed co-immunoprecipitation (coIP) assays from conditioned medium. Based on the qualitatively constant level of LRIM1-FLAG in the supernatant we can establish the qualitative level of expression/stability of the 6×His-tagged proteins as LRIM1, APL1C >> APL1B >> APL1A[1]; APL1A[2] not secreted. LRIM1 and APL1C are able to homodimerize *in vitro* [13], but heterodimerization is more efficient. In the case of LRIM1–6×His and LRIM1-FLAG, the 6×His-tagged homodimer is more efficiently formed and/or immunoprecipitated, such that LRIM1-FLAG did not co-immunoprecipiate with LRIM1–6×His in the conditions of our experiment (Fig. 7B). Despite its similarity to APL1A[1], APL1A[2] is not secreted from the cells [8] hence LRIM1-FLAG was not immunoprecipitated. However, LRIM1-FLAG co-immunoprecipitated with APL1A[1], APL1B and APL1C.

A further test for the formation of a specific heterodimer between LRIM1 and APL1B is whether an intermolecular disulfide bond is formed similar to LRIM1/APL1C [13]. Hence we

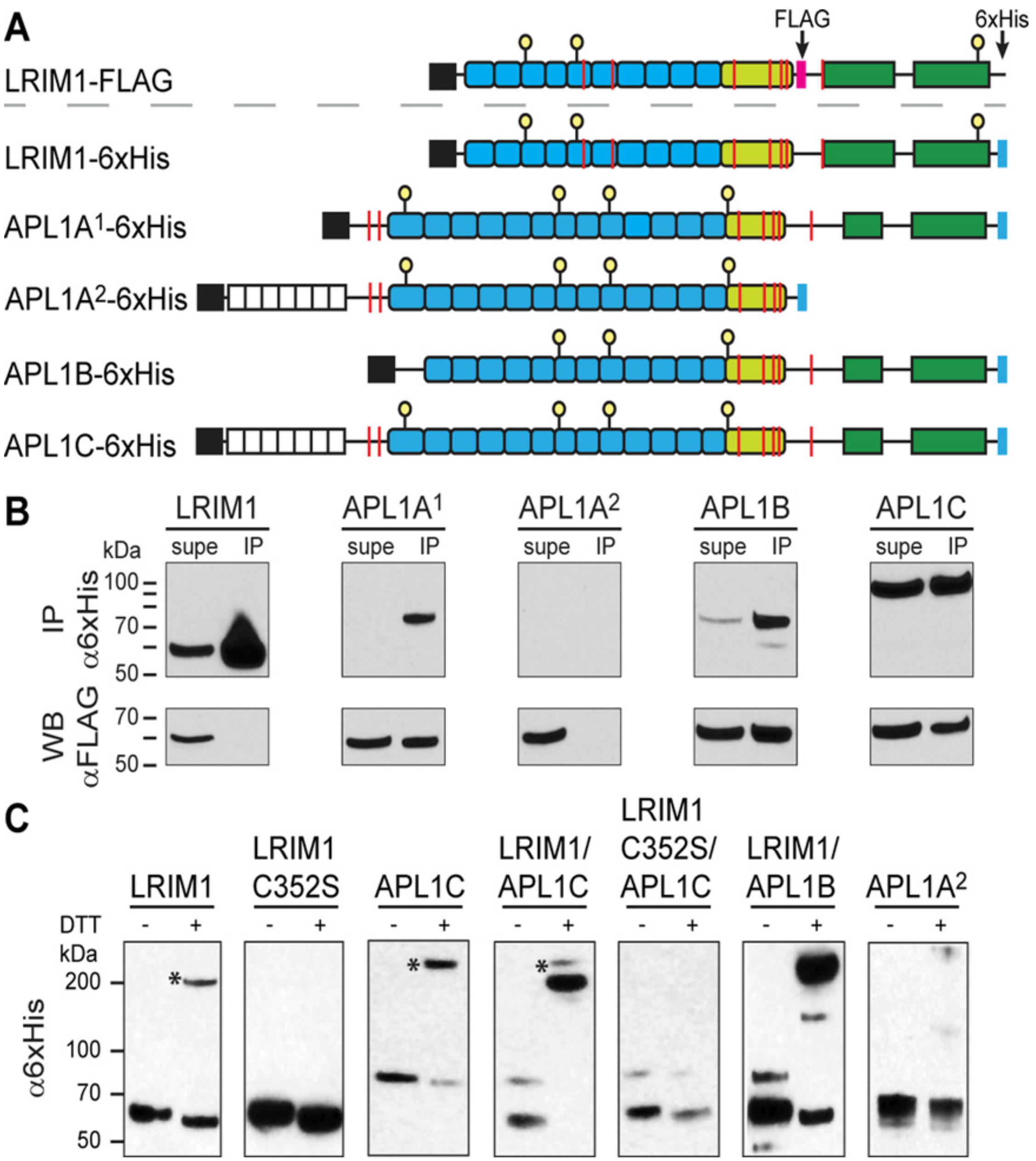

Fig 7. Disulfide-linked heterodimerization between full-length LRIM1 and APL1A—C. A: Schematic diagram showing the constructs that were used for the coIP experiment: signal peptide (black), low-complexity region (white), LRR domains (blue). Coiled-coil (green) FLAG tag (magenta), 6×His tag (cyan), cysteines (red lines). B: CoIP of FLAG-tagged LRIM1 with 6×His-tagged LRIM1, APL1A[1], APL1A[2], APL1B and APL1C. Western blots were performed with α6×His/HRP (top panel) and αFLAG/HRP (bottom panel) to detect the 6×His-tagged proteins and coIPed FLAG-tagged LRIM1, respectively. C: Conditioned media containing 6×His-tagged LRIM1, LRIM1-C352S, APL1C, APL1B and APL1A[2] (intracellular) were collected and evaluated with reducing (+DTT) or non-reducing (-DTT) SDS-PAGE and α6×His/HRP Western blotting.

doi:10.1371/journal.pone.0118911.g007

co-infected *T. ni* cells with 6×His-tagged LRIM1 and 6×His-tagged APL1A—C and analyzed reducing and non-reducing SDS-PAGE with Western blotting. Full-length LRIM1 and APL1C, but not LRIM1 C352S, form a heterodimer in the absence of reducing agent (Fig. 7C). Heterodimerization of LRIM1/APL1C is more efficient than homodimerization, but some LRIM1 and APL1C also form homodimers (marked with asterisk) in the absence of reducing agent. The

expression level of full-length APL1A[1]–6xHis was too low to confidently detect in this experiment, but LRIM1 and APL1B clearly formed a disulfide-mediated heterodimer.

Discussion

Protein complexes are essential for host innate immune responses to invasion by pathogens [35]. The LRR fold, commonly involved in protein-protein interactions [36], is not surprisingly involved in numerous central functions related to immunity and pathogenesis including cell signaling, platelet aggregation and attachment and invasion of pathogenic bacteria into host cells [37–44]. LRR domains consist of multiple repeats that form a curved solenoid structure with a parallel β-sheet on its concave face, which serves as the canonical binding surface. Numerous LRR proteins are known to form dimers and dimerization is almost always physiologically significant. Decorin, an extracellular matrix proteoglycan, forms a homodimer with an interface that spans over three quarters of the length of the concave face. Dimerization of decorin is coupled to protein folding and the interaction site is also involved in ligand binding [45]. The cell surface molecule AMIGO-1 exists as dimer with an interface of 1350 $Å^2$ formed by the concave side of the LRR domain as well as the LRRNT and LRRCT capping motifs. Dimerization of AMIGO-1 is necessary for proper cell surface expression and is likely to be involved in cell to cell adhesion [46]. Slits are multi-domain proteins containing four consecutive LRR domains that are important in neuronal development. The crystal structure of Slit2 showed that the protein exists as a homodimer where the entire concave face, including the C-terminal cap, is involved [47]. In contrast, Toll-like receptor (TLR) ectodomains dimerize via their lateral faces, forming an "m-shaped" structure [48]. Ligand-induced dimerization of TLRs is crucial for the recognition and initiation of innate immune responses against a wide variety of pathogens.

We have previously reported the heterodimeric structure of LRIM1 and APL1C, which is not mediated by an interaction between their respective LRR domains but mainly by a separate CC domain. Nevertheless, heterodimerization is also required for the function of LRIM1 and APL1C in stabilizing $TEP1_{cut}$. Here we describe the homodimer of APL1B, in which dimerization is mediated by domain-swapping of the leading LRR in the absence of a standard N-terminal capping motif. While domain swapping is a common mode of oligomerization in proteins [49], to our knowledge a domain-swapped oligomer of a LRR protein has not previously been reported. Despite the lack of a known functional role of APL1B within the *A. gambiae* immune system, we assert that homodimerization of APL1B is likely to be of physiological consequence.

The homodimerization of APL1B is due to the absence of a defined capping motif at the N-terminus, called the LRRNT. The unusual nature of this deficit was previously noted in a comprehensive sequence analysis of LRIM1 and APL1 proteins in the genomes of *A. gambiae*, *Aedes aegyptii* and *Culex quinquefasciatus* [14]. In the 83 proteins that were analyzed only five —*A. gambiae* LRIM1, APL1B and three LRIM20 orthologs—lacked the two-cysteine motif prior to the leading LRR that represents an LRRNT as found in the structure of APL1C-LRR [13]. To determine if any additional members may recently have been identified, we examined the N-terminal region of all 30 orthologs of APL1B (AGAP007035) listed in Vectorbase (www.vectorbase.org). A one-to-one ortholog for APL1B was not found and all of the orthologs that lack the LRRNT (nine proteins: AATE001420, ADIR007846, ADIR007869, AFAF007474, AFAF009836, AFAF016856, AFUN000279, AFUN000288, AFUN000597 from *A. atroparvus*, *A. dirus*, *A. farauti* and *A. funestus*) also lacked a signal peptide. Hence, the structure of APL1B may represent a unique feature within the LRIM1 family.

While the N-terminal sequence of APL1B is not conserved, the LRR that it binds to is. LRR III of APL1A and APL1C is generally conserved in long LRIM family members. We note that,

if APL1A or APL1C were truncated within LRR II, whether transcriptionally, post-translationally by an endopeptidase, or if APL1B was co-expressed with APL1A and APL1C, the N-terminus of APL1B could theoretically bind to LRR III in an equivalent manner as observed for the APL1B homodimer. Domain swapping is not only associated with oligomer assembly but also with misassembly [50]. Consider that intracellular retention of APL1A^2 is not readily explained (while being >90% identical to APL1C and APL1A^1, which are both secreted) and that at least some LRIM family proteins lacking a signal peptide are in fact intracellular and not simply misannotated. We speculate that domain swapping could occur during the co-expression of LRIM proteins and be associated with intracellular function or retention. Following secretion however, all evidence suggests that the LRR domains of LRIM1, APL1A^1 and APL1C are quite stable to proteolysis. Domain swapping of a folded LRR would therefore require specific activity of a chaperone or protease complex.

Full-length APL1B may not only form an N-terminal homodimer but is also capable of forming a heterodimer with LRIM1 via its CC domain. It should be noted that neither LRIM1 or APL1B homodimers nor LRIM1/APL1B heterodimers have been detected *in vivo*, and could reflect an artifact of heterologous expression. If an LRIM1/APL1B complex were formed it could potentially exist as a 2:2 heterotetramer. The CC domains of APL1B and APL1C are identical except for four point mutations within the HLH motif that is inserted within the CC structure, and the final ~30 residues of the coil (S1 Fig.). The successful coIP of APL1B with LRIM1 therefore suggests that this structural relationship is preserved within the LRIM1/APL1B complex along with the intermolecular disulfide bond.

We have not yet purified LRIM1/APL1B to homogeneity or demonstrated any interaction with TEP1, but hypothesize that the diversity in the CC domain serves to ensure the association of the correct N-terminal LRR domains (i.e. LRIM1 with APL1C) and that this diversity, specifically the HLH, may regulate the association with TEP1 or other TEPs (such as TEP3, TEP4, or TEP6). The structural diversity within the *APL1* genes is consistent with their being principal mediators of pathogen discrimination and specificity of the immune response to *Plasmodium*. TEP1 and LRIM1 in contrast are static structural components; variation in these proteins more likely affects affinity and reactivity, but not specificity, of the immune complex.

APL1A^1 diverges from APL1C to a greater extent than APL1B in the final ~60 residues of the CC domain, yet was observed to interact with LRIM1. Further *in vivo* studies are required to confirm the association of LRIM1 and APL1B. and APL1A^1. Our *in vitro* results are consistent with the observation that APL1A^2, which confers the most protective effect against *P. falciparum* and lacks a CC domain, is not constitutively secreted from cells [8]. Since APL1A^2 contains a signal peptide, the lack of secretion could be as a result of the absence of its binding partner in *T. ni* cells, incorrect protein folding or the absence of specific cofactors required for its secretion upon immune challenge *in vivo*. We hypothesize that APL1 heterodimers formed with LRIM1 exert their anti-*Plasmodium* activity via interaction with TEP1 (or other TEPs), but could also include TEP-independent pathways. No function has been attributed to the N-terminal low-complexity region but the absence of these repeats in APL1A^1 and APL1B and their presence in APL1A^2 and APL1C suggests that this region may confer a specific APL1 function and/or adaptive value [9].

In summary, the multivalent interactions identified for APL1B support a general model for the LRIM1/APL1 family as molecular scaffolds capable of generating a wide range of innate immune complexes. In the absence of an adaptive response, a genetically encoded repertoire of protein complexes and a high rate of polymorphism within immune genes can allow mosquito populations to adapt and defend against a diverse and rapidly evolving set of environmental pathogens. Understanding the molecular basis of this immune repertoire is important because the *Anopheles* immune defense to environmental pathogens is cross-reactive to human

pathogens such as *P. falciparum* of which it is a vector. *P. falciparum* itself is under constant selective pressure to adapt to and evade the *Anopheles* immune system. Future studies to determine the function of the *APL1* genes may lead to (i) genetic tests to accurately predict the vectoral capacity of field-sampled mosquitoes, and (ii) the development of strategies that enhance the natural immunity of *A. gambiae* to *Plasmodium*, thereby reducing transmission.

Materials and Methods

Alignment of APL1s

The Multalin and ESPript programs were used to generate S1 Fig. and 5 [51]. The gene IDs that were used for each protein are as follows: *APL1A* (AGAP007036), *APL1B* (AGAP007035), *APL1C* (AGAP007033), and *LRIM1* (AGAP006348).

Construct design

Full-length *LRIM1*, *APL1A[1]*, *APL1A[2]*, *APL1B* and *APL1C* (alleles from *A. gambiae* G3) were cloned into pFastBacI vectors (Invitrogen) with C-terminal 6×His tags. The LRR domains of *LRIM1* (residues 1–332), *APL1A[1]* (residues 1–439), *APL1B* (residues 1–370) and *APL1C* (residues 1–424 with deletion of residues 26–130 [13]) were also cloned into pFastBacI with C-terminal 6×His tags. To construct LRIM1-FLAG, a FLAG tag was inserted directly following the LRR domain (after LRRCT) by replacing the DRLIALKRK residues with DYKDDDDK. The LRIM1-FLAG and APL1A—C-6×His sequences were subcloned pFastBac-Dual to generate a dual-expressing recombinant baculovirus for coIP studies.

Protein expression and purification

All proteins were expressed using the Bac-to-Bac system (Invitrogen). *Spodoptera frugiperda* cells (Sf9, Invitrogen) in Sf900-III medium (Invitrogen) were used for the propagation of the baculoviruses while *Trichoplusia ni* cells (Expression Systems LLC) in ESF921 medium (Expression Systems LLC) were used for large-scale protein expression at 27°C. Infection with baculovirus constructs was performed at a multiplicity of infection (MOI) of 1.0. Harvesting of cells was optimized for each protein and ranged from 40–72 hours post infection (hpi). Purification and coIP experiments were performed as previously described [13,15,16]. The presence of disulfide-linked dimerization was detected by performing small-scale expression in *T.ni* cells infected with the full-length LRIM1 or APL1 viruses. Conditioned media were collected 24–72 hpi and evaluated with reducing or non-reducing 4–20% SDS-PAGE and α6×His/HRP (Clontech) or αFLAG-M2 (Sigma-Aldrich) Western blotting.

Purification of the LRR domains were performed as follows: following Talon affinity chromatography (Clontech) and elution in 250 mM NaCl, 20 mM Tris-HCl pH 7.8, 250 mM imidazole, the LRR domains were purified with MonoS ion exchange (GE) with Buffer A (50 mM NaCl, 20 mM HEPES pH 7.5) and a salt gradient with Buffer B (600 mM NaCl, 20 mM HEPES pH 7.5). The proteins were further purified with SEC on a Superdex 75 (16/600) column (GE) equilibrated with 100 mM NaCl, 20 mM HEPES pH 7.5.

SEC-MALLS and AUC

LRIM1-LRR, APL1A[1]-LRR, APL1B-LRR and APL1C-LRR proteins at 1 mg/ml were loaded on a Superdex 75 (10/300) column (GE) equilibrated with 100 mM NaCl, 20 mM HEPES pH 7.5. For SEC-MALLS, peaks were detected with an in-line UV detector (Jasco UV975) at 280 nm, a light scattering detector (DAWN EOS, Wyatt Technology Corp.) at 690 nm, and a refractive index detector (Optilab, Wyatt Technology Corp.). The molecular weight (MW) was

determined from the Debye plot of light scattering intensity versus scattering angle (Astra software, Wyatt Technology Corp.).

Sedimentation velocity was used to accurately determine the MW of APL1A[1]-LRR at a concentration of 0.7 mg/ml in a Beckman XL-1 centrifuge at 20°C and 45,000 rpm. Data was analyzed with SEDFIT [52].

Crystallization

Purified APL1B-LRR at 5 mg/ml rapidly crystallized in various conditions. Diffraction data to a resolution of 1.74 Å were collected from a crystal (space group $P2_12_12_1$) grown in 0.1 M $NaCH_3COO$ pH 5.2, 0.1 M Li_2SO_4, 1.0 M $(NH_4)_2PO_4$ at 293 K. The crystal was cryoprotected in the same condition containing 30% glycerol and frozen directly in a nitrogen cryostream at 100 K.

Data collection and refinement

X-ray data was collected at NSLS beam line X25C (Brookhaven National Laboratory). Data processing was performed with *HKL2000* [53]. Molecular replacement was performed with *MOLREP* [54] using APL1C-LRR (PDB ID 3O6N) as search model. Subsequent refinement was performed with *REFMAC* [55] followed by model building in *COOT* [56]. Side chain clashes were checked by the addition of riding hydrogens and geometric analysis by *MOLPROBITY* [57]. The total solvent-accessible surface area was analyzed by *PISA* [58]. The coordinates and structure factors are deposited in the PDB database with PDB ID 4XGO.

Small-angle x-ray scattering

SAXS data was collected at ALS SYBYLS beam line. Frozen aliquots of TEP1*R1, LRIM1/APL1C, APL1C-LRR and LRIM1-LRR were re-purified by SEC in PBS prior to data collection. Samples ranging from 1–5 mg/ml were interleaved with buffer and subjected to one second exposure. Buffer subtraction was performed by in-house software followed by primary data analysis using the *ATSAS* software package. Guinier analysis as a function of concentration confirmed each protein behaved as an ideal scatterer (R_G constant vs. concentration, I_0 linearly dependent with concentration and proportional to MW).

Three approaches to modeling the solution structure of each sample. First, a set of *ab initio* bead models were derived for each structure using *DAMMIF*, superimposed and compared to the crystal structure. Second, the crystal structure is used to model the solution scattering curve in CRYSOL. Third, we used the flexible fitting approach implemented by *ALLOSMOD-FOXS* to derive a best fit model incorporating missing residues and *N*-linked glycosylation from SAXS to the crystal structures of TEP1*R1 (4D94), LRIM1/APL1C (3OJA), APL1C-LRR (3O6N) and LRIM1-LRR (3O53) [13].

Supporting Information

S1 Fig. Structure-based sequence alignment of the full-length sequences of APL1A[1]. APL1A[2], APL1B, APL1C and LRIM1. The signal peptide sequences for each are shown in purple and do not form part of the alignment. Sequences that show similarities across a group are boxed in blue, similar residues across a group are boxed and highlighted yellow, similar residues within a group are in bold and residues that are strictly conserved are highlighted in red. Symbols above blocks of sequences correspond to the secondary structure of APL1C (Chain B) of PDB ID 3OJA containing helices (grey), beta sheets (black arrows), 3_{10}-helices (η) and turns (T). HLH residues are in cyan. Green numbers below the alignment indicate the presence of

disulphide bonds eg. the two number 1's form a bond. The green arrows indicate the Cys residues involved in intermolecular disulphide bond formation between the heterodimer LRIM1/APL1C.
(TIF)

S2 Fig. Primary data analysis of TEP1, LRIM1/APL1C, APL1C-LRR and LRIM1-LRR. Data shown as black line or points, error as grey vertical bars, fit to data shown as red line. (A) Guinier analysis ($\ln I$ *vs.* q^2) with line of best fit illustrated in fitting range. (B) I vs. q curve with fit by implicit Fourier Transform (*GNOM*). (C) Pairwise distribution function $p(r)$ derived by *GNOM*. (D) Kratky plot (Iq^2 *vs.* q). (E) Porod plot (Iq^4 *vs.* q).
(TIF)

S3 Fig. SAXS modeling of TEP1*R1, LRIM1-LRR, APL1C-LRR and LRIM1/APL1C. Superposition of *ab initio* SAXS models and best single model generated by *CRYSOL* for TEP1*R1, LRIM1/APL1C, APL1C-LRR and LRIM1-LRR SAXS data. (A) Bead model displayed as green surface, protein model by red/blue cartoon with CPK sticks for N-linked glycosylation. (B) Fit to experimental scattering curve for each of the static structural models shown above.
(TIF)

S4 Fig. Experimental verification of of oligomeric state of LRIM1 and APL1A—C LRR domains in solution. SEC-MALLS graphs show differential refactive index (dRI) on the left axis with blue trace, MW on right y axis. AUC shows fitting or raw sedimentation velocity data and transformed C(s) distribution from $0.1 < s < 6.1$. (A) LRIM1-LRR (SEC-MALLS), (B-C) APL1A[1] (AUC), (D) APL1B-LRR, (E) APL1C-LRR.
(TIF)

S5 Fig. APL1 polymorphisms mapped to structure. Mapping of the polymorphisms (red) between APL1A-C onto APL1C-LRR shown as a grey surface structure (PDB ID 3O6N).
(TIF)

Acknowledgments

The authors thank Prof. Kenneth Vernick for helpful discussions as well as originally supplying sequences for G3 alleles of *APL1A[1]*, *APL1A[2]* and *APL1B*. The authors would also like to thank Michael J. Robertson for helping with the initial studies on the APL1A[1] protein and Bill Eliason for the SEC-MALLS experiments. SAXS experiments were conducted at the Advanced Light Source (ALS), a national user facility operated by Lawrence Berkeley National Laboratory on behalf of the Department of Energy, Office of Basic Energy Sciences, through the Integrated Diffraction Analysis Technologies (IDAT) program, supported by DOE Office of Biological and Environmental Research. Diffraction data of APL1B-LRR were collected at National Synchrotron Light Source (NSLS) beam line X25C (Brookhaven National Laboratory). Use of the NSLS was supported by the U.S. Department of Energy, Office of Science, Office of Basic Energy Sciences, under Contract No. DE-AC02–98CH10886. Additional support comes from the National Institute of Health project MINOS (R01GM105404).

Author Contributions

Conceived and designed the experiments: MW RHGB. Performed the experiments: MW BJS. Analyzed the data: MW BJS RHGB. Wrote the paper: MW RHGB.

References

1. WHO World Malaria Report 2014. 2014. Geneva, Switzerland: World Health Organization. 242 p.

2. Dong Y, Aguilar R, Xi Z, Warr E, Mongin E, Dimopoulos G. *Anopheles gambiae* immune responses to human and rodent *Plasmodium* parasite species. PLoS Pathog 2006; 2: e52. PMID: 16789837
3. Frolet C, Thoma M, Blandin S, Hoffmann JA, Levashina EA. Boosting NF-κB-dependent basal immunity of *Anopheles gambiae* aborts development of *Plasmodium berghei*. Immunity 2006; 25: 677–685. PMID: 17045818
4. Niare O, Markianos K, Volz J, Oduol F, Toure A, Bagayoko M, et al. Genetic loci affecting resistance to human malaria parasites in a West African mosquito vector population. Science 2002; 298: 213–216. PMID: 12364806
5. Riehle MM, Kyriacos M, Niaré O, Xu J, Li J, Touré AM, et al. Natural malaria infection in *Anopheles gambiae* is regulated by a single genomic control region. Science 2006; 312: 577–579. PMID: 16645095
6. Riehle MM, Markianos K, Lambrechts L, Xia A, Sharakhov I, Koella JC, et al. A major genetic locus controlling natural *Plasmodium falciparum* infection is shared by East and West African *Anopheles gambiae*. Malar J 2007; 6: 87. PMID: 17612409
7. Riehle MM, Xu J, Lazzaro BP, Rottschaefer SM, Coulibaly B, Sacko M, et al. *Anopheles gambiae* APL1 is a family of variable LRR proteins required for Rel1-mediated protection from the malaria parasite, *Plasmodium berghei*. PLoS One 2008; 3: e3672. doi: 10.1371/journal.pone.0003672 PMID: 18989366
8. Holm I, Lavazec C, Garnier T, Mitri C, Riehle MM, Bischoff E, et al. Diverged alleles of the *Anopheles gambiae* leucine-rich repeat gene APL1A display distinct protective profiles against *Plasmodium falciparum*. PLoS One 2012; 7: e52684. doi: 10.1371/journal.pone.0052684 PMID: 23285147
9. Rottschaefer SM, Riehle MM, Coulibaly B, Sacko M, Niare O, Morlais I, et al. Exceptional diversity, maintenance of polymorphism, and recent directional selection on the APL1 malaria resistance genes of *Anopheles gambiae*. PLoS Biol 2011; 9: e1000600. doi: 10.1371/journal.pbio.1000600 PMID: 21408087
10. Osta MA, Christophides GK, Kafatos FC. Effects of mosquito genes on *Plasmodium* development. Science 2004; 303: 2030–2032. PMID: 15044804
11. Fraiture M, Baxter RHG, Steinert S, Chelliah Y, Frolet C, Quispe-Tintaya W, et al. Two mosquito LRR proteins function as complement control factors in the TEP1-mediated killing of *Plasmodium*. Cell Host Microbe 2009; 5: 273–284. doi: 10.1016/j.chom.2009.01.005 PMID: 19286136
12. Povelones M, Waterhouse RM, Kafatos FC, Christophides GK. Leucine-rich repeat protein complex activates mosquito complement in defense against *Plasmodium* parasites. Science 2009; 324: 258–261. doi: 10.1126/science.1171400 PMID: 19264986
13. Baxter RHG, Steinert S, Chelliah Y, Volohonsky G, Levashina EA, Deisenhofer J. A heterodimeric complex of the LRR proteins LRIM1 and APL1C regulates complement-like immunity in *Anopheles gambiae*. Proc Natl Acad Sci USA 2010; 107: 16817–16822. doi: 10.1073/pnas.1010575107 PMID: 20826443
14. Waterhouse RM, Povelones M, Christophides GK. Sequence-structure-function relations of the mosquito leucine-rich repeat immune proteins. BMC Genomics 2010; 11: 531. doi: 10.1186/1471-2164-11-531 PMID: 20920294
15. Baxter RHG, Chang C-I, Chelliah Y, Blandin S, Levashina EA, Deisenhofer J. Structural basis for conserved complement factor-like function in the antimalarial protein TEP1. Proc Natl Acad Sci USA 2007; 104: 11615–11620. PMID: 17606907
16. Le BV, Williams M, Logarajah S, Baxter RHG. Molecular basis for genetic resistance of *Anopheles gambiae* to *Plasmodium*: structural analysis of TEP1 susceptible and resistant alleles. PLoS Pathog 2012; 8: e1002958. doi: 10.1371/journal.ppat.1002958 PMID: 23055931
17. Levashina EA, Moita LF, Blandin S, Vriend G, Lagueux M, Kafatos FC. Conserved role of a complement-like protein in phagocytosis revealed by dsRNA knockout in cultured cells of the mosquito, *Anopheles gambiae*. Cell 2001; 104: 709–718. PMID: 11257225
18. Blandin S, Levashina EA. Reverse genetics analysis of antiparasitic responses in the malaria vector, Anopheles gambiae. Methods Mol Biol 2008; 415: 365–377. doi: 10.1007/978-1-59745-570-1_21 PMID: 18370165
19. Blandin SA, Wang-Sattler R, Lamacchia M, Gagneur J, Lycett G, Ning Y, et al. Dissecting the genetic basis of resistance to malaria parasites in *Anopheles gambiae*. Science 2009; 326: 147–150. doi: 10.1126/science.1175241 PMID: 19797663
20. White BJ, Lawniczak MKN, Cheng C, Coulibaly MB, Wilson MD, Sagnon NF, et al. Adaptive divergence between incipient species of *Anopheles gambiae* increases resistance to *Plasmodium*. Proc Natl Acad Sci USA 2011; 108: 244–249. doi: 10.1073/pnas.1013648108 PMID: 21173248
21. Molina-Cruz A, DeJong RJ, Ortega C, Haile A, Abban E, Rodrigues J, et al. Some strains of *Plasmodium falciparum*, a human malaria parasite, evade the complement-like system of *Anopheles gambiae*

mosquitoes. Proc Natl Acad Sci USA 2012; 109: E1957–1962. doi: 10.1073/pnas.1121183109 PMID: 22623529

22. Collins FH, Sakai RK, Vernick KD, Paskewitz S, Seeley DC, Miller LH, et al. Genetic selection of a *Plasmodium*-refractory strain of the malaria vector *Anopheles gambiae*. Science 1986; 234: 607–610. PMID: 3532325
23. Harris C, Lambrechts L, Rousset F, Abate L, Nsango SE, Fontenille D, et al. Polymorphisms in *Anopheles gambiae* immune genes associated with natural resistance to *Plasmodium falciparum*. PLoS Pathog 2010; 6: e1001112. doi: 10.1371/journal.ppat.1001112 PMID: 20862317
24. Mitri C, Jacques J-C, Thiery I, Riehle MM, Xu J, Bischoff E, et al. Fine pathogen discrimination within the APL1 gene family protects *Anopheles gambiae* against human and rodent malaria species. PLoS Pathog 2009; 5: e1000576. doi: 10.1371/journal.ppat.1000576 PMID: 19750215
25. Hurd H, Taylor PJ, Adams D, Underhill A, Eggleston P. Evaluating the costs of mosquito resistance to malaria parasites. Evolution 2005; 59: 2560–2572. PMID: 16526504
26. Garver LS, Bahia AC, Das S, Souza-Neto JA, Shiao J, Dong Y, et al. *Anopheles* Imd pathway factors and effectors in infection intensity-dependent anti-Plasmodium action. PLoS Pathog 2012; 8: e1002737. doi: 10.1371/journal.ppat.1002737 PMID: 22685401
27. Molina-Cruz A, Garver LS, Alabaster A, Bangiolo L, Haile A, Winikor J, et al. The human malaria parasite Pfs47 gene mediates evasion of the mosquito immune system. Science 2013; 340: 984–987. doi: 10.1126/science.1235264 PMID: 23661646
28. Bella J, Hindle KL, McEwan PA, Lovell SC. The leucine-rich repeat structure. Cell Mol Life Sci 2008; 65: 2307–2333. doi: 10.1007/s00018-008-8019-0 PMID: 18408889
29. Povelones M, Upton LM, Sala KA, Christophides GK. Structure-function analysis of the *Anopheles gambiae* LRIM1/APL1C complex and its interaction with complement C3-like protein TEP1. PLoS Pathog 2011; 7: e1002023. doi: 10.1371/journal.ppat.1002023 PMID: 21533217
30. Svergun DI. Determination of the regularization parameter in indirect-transform methods using perceptual criteria. J Appl Cryst 1992; 25: 495–503.
31. Franke DA, Svergun DI. DAMMIF, a program for rapid ab-initio shape determination in small-angle scattering. J Appl Cryst 2009; 42: 342–346.
32. Svergun DI, Barberato C, Koch MHJ. CRYSOL—A program to evaluate x-ray solution scattering of biological macromolecules from atomic coordinates. J Appl Cryst 1995; 28: 768–773.
33. Guttman M, Weinkam P, Sali A, Lee KK. All-atom ensemble modeling to analyze small-angle x-ray scattering of glycosylated proteins. Structure 2013; 21: 321–331. doi: 10.1016/j.str.2013.02.004 PMID: 23473666
34. Bennett MJ, Schlunegger MP, Eisenberg D. 3D domain swapping: a mechanism for oligomer assembly. Protein Sci 1995; 4: 2455–2468. PMID: 8580836
35. Rebsamen M, Kandasamy RK, Superti-Furga G. Protein interaction networks in innate immunity. Trends Immunol 2013; 34: 610–619. doi: 10.1016/j.it.2013.05.002 PMID: 23827258
36. Kobe B, Deisenhofer J. The leucine-rich repeat: a versatile binding motif. Trends Biochem Sci 1994; 19: 415–421. PMID: 7817399
37. Kresse H, Schönherr E. Proteoglycans of the extracellular matrix and growth control. J Cell Physiol 2001; 189: 266–274. PMID: 11748584
38. Niemann HH, Schubert W-D, Heinz DW. Adhesins and invasins of pathogenic bacteria: a structural view. Microbes Infect 2004; 6: 101–112. PMID: 14738899
39. Andrews RK, Berndt MC. Platelet physiology and thrombosis. Thromb Res 2004; 114: 447–453. PMID: 15507277
40. Matilla A, Radrizzani M. The Anp32 family of proteins containing leucine-rich repeats. Cerebellum 2005; 4: 7–18. PMID: 15895553
41. Hohenester E, Hussain S-A, Howitt JA. Interaction of the guidance molecule Slit with cellular receptors. Biochem Soc Trans 2006; 34: 418–421. PMID: 16709176
42. West AP, Koblansky AA, Ghosh S. Recognition and signaling by toll-like receptors. Annual Review of Cell and Developmental Biology 2006; 22: 409–437. PMID: 16822173
43. Bierne H, Sabet C, Personnic N, Cossart P. Internalins: a complex family of leucine-rich repeat-containing proteins in *Listeria monocytogenes*. Microbes Infect 2007; 9: 1156–1166. PMID: 17764999
44. Gay NJ, Gangloff M. Structure and function of Toll receptors and their ligands. Ann Rev Biochem 2007; 76: 141–165. PMID: 17362201
45. Scott PG, McEwan PA, Dodd CM, Bergmann EM, Bishop PN, Bella J. Crystal structure of the dimeric protein core of decorin, the archetypal small leucine-rich repeat proteoglycan. Proc Natl Acad Sci USA 2004; 101: 15633–15638. PMID: 15501918

46. Kajander T, Kuja-Panula J, Rauvala H, Goldman A. Crystal structure and role of glycans and dimerization in folding of neuronal leucine-rich repeat protein AMIGO-1. J Mol Biol 2011; 413: 1001–1015. doi: 10.1016/j.jmb.2011.09.032 PMID: 21983541

47. Seiradake E, von Philipsborn AC, Henry M, Fritz M, Lortat-Jacob H, Jamin M, et al. Structure and functional relevance of the Slit2 homodimerization domain. EMBO Rep 2009; 10: 736–741. doi: 10.1038/embor.2009.95 PMID: 19498462

48. Botos I, Segal DM, Davies DR. The structural biology of Toll-like receptors. Structure 2011; 19: 447–459. doi: 10.1016/j.str.2011.02.004 PMID: 21481769

49. Rousseau F, Schymkowitz J, Itzhaki LS. Implications of 3D domain swapping for protein folding, misfolding and function. Adv Exp Med Biol 2012; 747: 137–152. doi: 10.1007/978-1-4614-3229-6_9 PMID: 22949116

50. Schlunegger MP, Bennett MJ, Eisenberg D. Oligomer formation by 3D domain swapping: a model for protein assembly and misassembly. Adv Protein Chem 1997; 50: 61–122. PMID: 9338079

51. Gouet P, Courcelle E, Stuart DI, Metoz F. ESPript: analysis of multiple sequence alignments in PostScript. Bioinformatics 1999; 15: 305–308. PMID: 10320398

52. Schuck P. Size-distribution analysis of macromolecules by sedimentation velocity ultracentrifugation and lamm equation modeling. Biophys J 2000; 78: 1606–1619. PMID: 10692345

53. Otwinowski Z, Minor W. Processing of x-ray diffraction data collected in oscillation mode. Methods Enzymol 1997; 276: 307–326.

54. Vagin AA, Teplyakov A. MOLREP: an automated program for molecular replacement. J Appl Cryst 1997; 30: 1022–1025.

55. Murshudov GN, Vagin AA, Dodson EJ. Refinement of macromolecular structures by the maximum-likelihood method. Acta Crystallographica Section D: Biological crystallography 1997; 53: 240–255. PMID: 15299926

56. Emsley P, Lohkamp B, Scott WG, Cowtan K. Features and development of *Coot*. Acta Crystallogr D Biol Crystallogr 2010; 66: 486–501. doi: 10.1107/S0907444910007493 PMID: 20383002

57. Chen VB, Arendall WBr, Headd JJ, Keedy DA, Immormino RM, Kapral GJ, et al. *MolProbity*: all-atom structure validation for macromolecular crystallography. Acta Crystallogr D Biol Crystallogr 2010; 66: 12–21. doi: 10.1107/S0907444909042073 PMID: 20057044

58. Krissinel E, Henrick K. Inference of macromolecular assemblies from crystalline state. J Mol Biol 2007; 372: 774–797. PMID: 17681537
